# Deep clustering representation of spatially resolved transcriptomics data using multi-view variational graph auto-encoders with consensus clustering

**DOI:** 10.1016/j.csbj.2024.11.041

**Published:** 2024-12-02

**Authors:** Jinyun Niu, Fangfang Zhu, Taosheng Xu, Shunfang Wang, Wenwen Min

**Affiliations:** a School of Information Science and Engineering, Yunnan University, Kunming, 650091, Yunnan, China; b School of Health and Nursing, Yunnan Open University, Kunming, 650599, Yunnan, China; c Hefei Institutes of Physical Science, Chinese Academy of Sciences, Hefei, 230031, Anhui, China

**Keywords:** Spatially resolved transcriptomics, Deep learning, Multi-view variational graph autoencoders, Consensus clustering

## Abstract

The rapid development of spatial transcriptomics (ST) technology has provided unprecedented opportunities to understand tissue relationships and functions within specific spatial contexts. Accurate identification of spatial domains is crucial for downstream spatial transcriptomics analysis. However, effectively combining gene expression data, histological images and spatial coordinate data to identify spatial domains remains a challenge. To this end, we propose STMVGAE, a novel spatial transcriptomics analysis tool that combines a multi-view variational graph autoencoder with a consensus clustering framework. STMVGAE begins by extracting histological images features using a pre-trained convolutional neural network (CNN) and integrates these features with gene expression data to generate augmented gene expression profiles. Subsequently, multiple graphs (views) are constructed using various similarity measures, capturing different aspects of the spatial and transcriptional relationships. These views, combined with the augmented gene expression data, are then processed through variational graph auto-encoders (VGAEs) to learn multiple low-dimensional latent embeddings. Finally, the model employs a consensus clustering method to integrate the clustering results derived from these embeddings, significantly improving clustering accuracy and stability. We applied STMVGAE to five real datasets and compared it with five state-of-the-art methods, showing that STMVGAE consistently achieves competitive results. We assessed its capabilities in spatial domain identification and evaluated its performance across various downstream tasks, including UMAP visualization, PAGA trajectory inference, spatially variable gene (SVG) identification, denoising, batch integration, and other analyses. All code and public datasets used in this paper is available at https://github.com/wenwenmin/STMVGAE and https://zenodo.org/records/13119867.

## Graphical abstract

**Figure:**
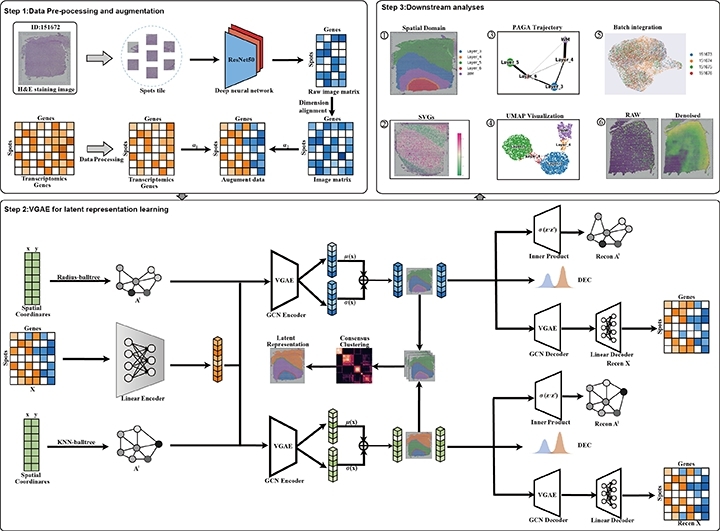

## 1 Introduction

The tissues of living organisms comprise various cell types, each with distinct functions. Complex tissues and different cell types are closely related to spatial distribution [1]. Combining spatial location data with gene expression profiles enables researchers to conduct more detailed spatial transcriptome analyses [2]. In recent years, spatial transcriptomics has witnessed the emergence of several breakthrough technologies, including 10x Visium [3], Slide-seq [4], [5], Stereo-seq [6], PIXEL-seq [7], and High Definition Spatial Transcriptome (HDST) [8]. These methods capture gene expression profiles at multiple cellular or even subcellular levels at specific locations. By capturing extensive gene expression profiles corresponding to spatial positions, these methods enable researchers to analyze spatial transcriptomics data more accurately, facilitating a deeper understanding of tissue function and cell structure.

To comprehensively analyze ST data, the task of spatial domain identification is crucial. Spatial domain identification is essentially a clustering task aimed at accurately assigning domain labels to captured spots or cells using ST data. The current methods for identifying spatial domains are divided into the following two categories. First, early spatial domain identification methods are traditional clustering methods such as K-means and Louvain [9]. Specifically, they first reduce the feature dimension through methods such as PCA, t-SNE [10] or Uniform Manifold Approximation and Projection (UMAP) [11], and then K-means or Louvain is used to cluster. However, these methods only utilize gene expression profiles for clustering and do not combine histological images and spatial location data. As a result, they may lead to discontinuity in the identified spatial domains. BayesSpace [12] is a method based on Bayesian statistics, that utilizes spatial prior knowledge to encourage adjacent points to belong to the same cluster, thereby achieving spatial clustering. Giotto [13], which employs the Hidden Markov Random Field (HMRF) model to detect spatial domains based on the positional relationship between spots. stLearn [14] constructs spatial location information based on gene expression features and smoothly embeds this information into low-dimensional spatial expression data for spatial domain identification. BASS [15] uses a Bayesian hierarchical modeling framework for clustering analysis in spatial transcriptomics, which facilitates multi-scale and multi-sample analysis.

Secondly, graph convolutional neural networks have shown great potential in the fields of unstructured data and relationship modeling in recent years, and several excellent methods have emerged for spatial domain identification [16]. SpaGCN [17] utilizes histological images information to construct three-dimensional spatial distances and combines this with a neural network that includes a self-supervised module for training. STAGATE [18] employs an autoencoder with a graph attention mechanism to aggregate spatial information and gene expression data for identifying spatial domains. SEDR [19] uses a variational graph autoencoder with a masking mechanism to incorporate spatial neighborhood relationships into the spots. DeepST [20] constructs histological images information into domain relationships to enhance gene expression and optimize spatial domain identification. GraphST [21] introduces graph comparison learning into spatial domain recognition tasks and integrates it with graph convolutional neural networks. STMGCN [22] represents graph structure information by constructing various adjacency matrices and employs an attention mechanism to integrate the low-dimensional representations obtained by a deep network with a self-supervised module. STAMaker [23] integrates the clustering results obtained from multiple STAGATEs through consensus clustering as labels for subsequent tasks. conST [24] utilizes contrastive learning to integrate gene expression, spatial information, and histological images data. It applies data augmentation and uses three levels of contrastive learning to minimize or maximize the mutual information between different embeddings, ultimately learning low-dimensional representations. Stardust [25] is an innovative spatial transcriptomics clustering method that integrates gene expression, spatial information, and histological images through a dynamic space-aware modularity optimization approach.

A detailed summary of the comparison methods is provided in Supplementary Table S1.

The above methods have contributed to improvements in the spatial domain identification task from different aspects, but several limitations remain. Firstly, most existing methods fail to fully utilize histological images, which limits their ability to accurately identify spatial domains. Histological images contain valuable information about tissue structure and cell organization, providing additional spatial context that is not captured by gene expression data alone [14]. Secondly, many existing methods rely solely on spatial coordinate information to construct a single view. When training is conducted using such a single view, the spots within the ST data lack domain affinity, meaning they cannot effectively integrate neighboring information [22]. Finally, integrating the clustering outputs from multi-view networks that contain different structural information poses a significant challenge.

To solve the above problems, we propose STMVGAE, a consensus clustering framework [26] that utilizes multi-view networks to accurately identify spatial domains. Specifically, we use a pre-trained convolutional neural network (CNN) to extract detailed information from histological images tiles, which is then fused with gene expression profiles to create an enhanced gene expression matrix. Histological images contain critical information about tissue structure, cell morphology, and the spatial distribution of spots. This added spatial context helps correct potential biases or inaccuracies in gene expression measurements, partially mitigating the sparsity of spatial transcriptomics (ST) data, ultimately leading to more reliable results [20]. Next, we construct multiple views using different methods. The enhanced gene expression profile and these multiple views serve as inputs for variational graph autoencoders (VGAEs), which are trained to obtain embeddings. This approach provides a more comprehensive understanding of the data. By considering multiple views or perspectives of the same biological phenomenon, such as gene expression profiles and spatial location information, we can capture a broader range of information. Each view may highlight different aspects or characteristics of the data [27]. Finally, Mclust [28] is used to cluster the embeddings and generate multiple clustering outputs. We apply a consensus clustering strategy to integrate these outputs into a unified consensus clustering label for spatial domain identification. Consensus clustering has been widely used in bulk and scRNA-seq transcriptomics [29], [30], showing great potential in enhancing the stability and robustness of the results by integrating multiple clustering results. We have innovatively introduced consensus clustering into ST data analysis, addressing the lack of a consensus clustering framework in this field. STMVGAE accurately identifies spatial domains by integrating different ST data and supports a variety of downstream tasks, including UMAP [11] visualization, PAGA [31] trajectory inference, denoising, and batch integration. We applied STMVGAE to five real datasets from different platforms, and our method consistently achieved competitive results compared to state-of-the-art approaches.

## 2 Materials and methods

### 2.1 Datasets and data preprocessing

In this section, we aimed to assess the effectiveness of STMVGAE by utilizing five real datasets sourced from different platforms, including 10x Genomics [3], Stereo-seq [4], and Spatial Research ST platforms (Table 1).

**Table 1:** The statistics of the datasets.

Datasets	Spots	Genes	Slices	Domain	Platforms
DLPFC	3460-4789	33538	12	5-7	10x Genomics
BCDC	2518	17943	1	2	10x Genomics
Melanoma	293	16148	1	4	Spatial Research
BRCA	3798	36601	1	20	10x Genomics
Olfactory	19109	27106	1	-	Stereo-seq

Firstly, we examined the human dorsolateral prefrontal cortex (DLPFC) dataset containing 12 slices. Each slice in this dataset consists of six cortical layers and one white matter (WM) layer. The number of spots in each slice ranges from 3460 to 4789, and the number of genes is 33,538. The original author Maynard et al. [32] annotated each spot according to the hierarchical structure. The dataset was measured by the 10x Genomics platform and can be downloaded in its entirety at SpatialLIBD. Additionally, since this dataset was sequenced through Visium technology, each spot had six adjacent nodes and formed a regular hexagon.

For the second dataset, we selected the human breast cancer: ductal carcinoma in situ (BCDC) published by the 10x Genomics platform. This data contains a piece of ductal carcinoma tissue, which includes healthy areas and cancer-spread areas. It contains a total of 2518 spots. We obtained manual annotation of the dataset from Ni et al. [33], who divided the data into cancer and non-cancerous regions.

In the third dataset, we analyzed the human melanoma cancer dataset (Melanoma), which was manually annotated by Thrane et al. [34]. We analyzed the second replicate of biopsy 1 that had manual annotation in the data. Biopsy 1 contains 293 spots and 16146 genes, and the data is divided into 5 different regions.

We used another human breast cancer (BRCA) dataset published by the 10x Genomics platform for the fourth dataset. We obtained the raw data and manual annotation from Fu et al. [19], and the authors of SEDR annotated it based on H&E staining images and hierarchical structures.

The last dataset came from the mouse olfactory bulb cell dataset (Olfactory) on the Stereo-seq platform. The data author did not manually label each spots, but divided the different layers of the data according to the laminar structure. Details of all datasets we used are provided in the Supplementary Table S2.

All five real ST datasets underwent the same data preprocessing steps. First, genes expressed in fewer than 50 cells/spots were removed. Then, gene expression or the enhanced gene expression was log-transformed using the SCANPY package [35] and normalized based on library size. Finally, 3,000 highly variable genes were selected as input for STMVGAE.

### 2.2 Overview of STMVGAE

**Fig. 1:**
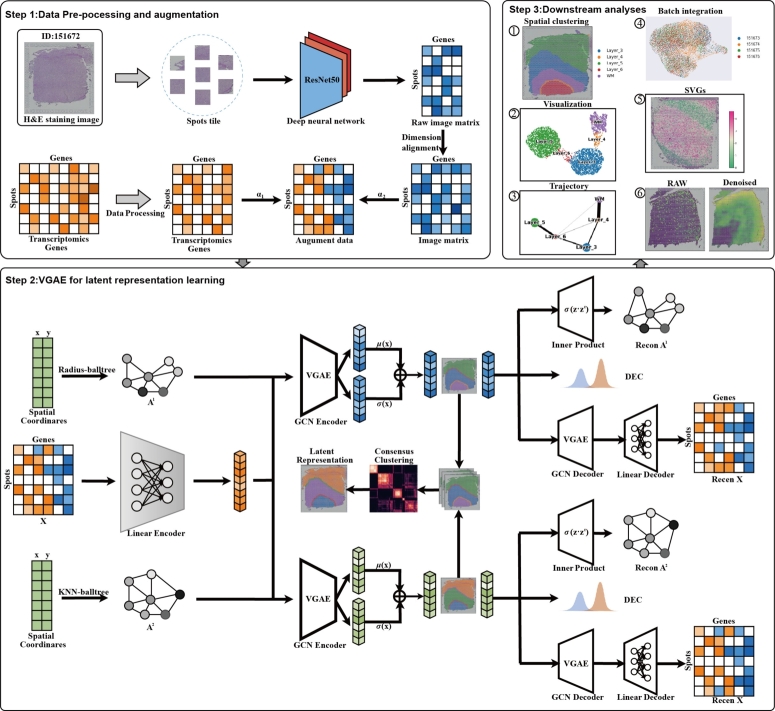
Workflow of STMVGAE. 1.STMVGAE first extracts the high-dimensional information of tissue morphology through CNN and integrates it into gene expression to form the augmented gene expression. 2.STMVGAE then uses different construction ideas to construct multi-view adjacency matrices. For each view/graph, graph convolution is applied to embed a single view into the augmented gene expressions, resulting in view-specific low-dimensional embedding representations. We cluster the multiple low-dimensional embedding representations obtained, and unify these clustering results with consensus clustering, and finally obtain the assignment results of spots. 3.STMVGAE can perform a variety of downstream tasks, including spatial domain identification, UMAP visualization, PAGA trajectory inference, spatially variable genes (SVGs) identification, denoising, and batch integration. Our downstream task analysis is performed on the embeddings generated by the Radius-balltree view.

STMVGAE is a consensus clustering framework for integrating multi-view clustering outcomes. The overall workflow of STMVGAE is shown in Fig. 1. STMVGAE extracts histological images features based on spatial location, and integrate them with gene expression data. Histological images provide rich information on cell morphology and structure, which helps to more accurately identify and locate cells, thereby improving the accuracy of interpreting gene expression data. At the same time, in order to fully characterize the relationship between spots, we construct multiple views using different data structures, we also try to use gene expression data to calculate the Spearman coefficient and Cosine similarity to construct the views and evaluate the performance of STMVAGE. STMVGAE then takes the enhanced gene expression and multiple views as input to learning view-specific latent embeddings $Z^{\left(i\right)}$, where $Z^{\left(i\right)}$ represents the *i*-th low-dimensional latent embedding generated by the *i*-th view and the enhanced gene expression. Finally, Mclust [28] is used to cluster $Z^{\left(i\right)}$ to obtain the prediction spot assignment $Y^{\left(i\right)}$ and we introduce consensus clustering to integrate $Y^{\left(1\right)},Y^{\left(2\right)},...,Y^{\left(M\right)}$ to generate the final consensus clustering result $Y^{\left(⁎\right)}$. In addition, for the sake of the introduction and beauty of Fig. 1, we only show the process of constructing $A^{1}$ and $A^{2}$ during training. We provide more diverse graph training STMVGAE.

### 2.3 ST data augumentation

Spatial transcriptome sequencing technology provides us with information on gene expression profiles and spatial coordinates. Spatial transcriptomics data often faces the issue of data sparsity, especially in high-dimensional gene expression data, where many spatial locations may have missing or noisy gene expression values. By incorporating histological images features, additional stable spatial information can be provided to the model, helping to fill in these gaps and uncertainties, thereby mitigating the impact of data sparsity. This supplementary information helps improve the performance of tasks such as clustering and classification.

For ST data with histological images, we initially segment the image (spots tile) based on the spatial coordinates of each spot. When enhancing gene expression, the spatial information of the histological images patches must first be aligned with the spatial coordinates of the gene expression data. This alignment is not only to ensure that each part of the histological images corresponds to the appropriate location in the gene expression matrix, but also to ensure the validity of subsequent analyses. We utilize the *torchvision.transforms* function to process and enhance the partial images, including normalization and auto-contrasting, etc. Following this, we employ a pre-trained convolutional neural network (optional, default is ResNet50) from the *torchvision.models* function to extract 1000-dimensional raw image features as $H^{\left(0\right)}$. We construct an autoencoder with two fully connected layers for the encoder and a symmetric decoder to perform dimensionality expansion on the histological images features, aligning them with the dimensions of gene expression data. The model is optimized through reconstruction loss. This approach allows for flexible expansion of input features to higher dimensions, providing richer features for subsequent analysis. The formulas for the fully connected layers are as follows:

(1) $$MS=\text{Linear}(H^{\left(l-1\right)})=\text{ReLU}(\text{BN}(W^{\left(l\right)}H^{\left(l-1\right)}+b^{\left(l\right)}))$$

where *W* is the learnable parameter, *b* is the bias term, and *MS* is the image feature matrix after data alignment, the dimension of *MS* is consistent with the preprocessed gene expression data *GE*.

The enhanced gene expression data $\overset{˜}{GE}$ is obtained by adding *MS* and *GE* with different weights, and $\overset{˜}{GE}$ is used as the input to STMVGAE:

(2) $$\overset{˜}{GE}=\alpha_{1}⁎MS+\alpha_{2}⁎GE$$

### 2.4 Spatial graph construction

To merge neighbor information into given spots, we convert spatial location into an undirected adjacency matrix. First, we predefine a radius *r*, if the Euclidean distance between spot *j* and a given spot *i* is less than *r*, then $A_{ij}=A_{ji}=1$. We then use the k-nearest neighbor (KNN) method to calculate the *k* nearest neighbors to a given spot *i* to construct an adjacency matrix. The above two methods construct the adjacency matrix from two different perspectives: directly selecting the nearest neighbor and selecting the nearest neighbor by radius. Moreover, considering the use of different data structure methods for construction, we adopt two different data structures, Balltree and Kdtree, provided by the sklearn package [36]. Based on the above ideas, we construct four adjacency matrices, namely Radius-balltree, Radius-kdtree, KNN-balltree, and KNN-kdtree, record as $A^{\left(1\right)},A^{\left(2\right)},A^{\left(3\right)}$, and $A^{\left(4\right)}$.

### 2.5 Gene graph construction

To further represent the underlying spatial relationship from different perspectives, we take the gene expression matrix to construct an adjacency matrix [29]. Specifically, we use the Spearman coefficient and Cosine similarity respectively to calculate the values between a given spot *i* and all other spots, and then select the k spots with the largest values as the neighbors of the given spots *i*, then $A_{ij}=A_{ji}=1$. The Spearman coefficient and cosine similarity are calculated as follows:

(3) $$Spearman(x_{i},x_{j})=1-\frac{6\sum_{i+1}^{n}d_{i}^{2}}{n(n^{2}-1)}$$

where $d_{i}$ is the grade difference between spots *i* and spot *j*, and *n* is the number of spots, $x_{i}$ and $x_{j}$ represent the features corresponding to spot *i* and spot *j* in the gene expression matrix $X\in R^{N\times N}$.

(4) $$cos(x_{i},x_{j})=\frac{x_{i}\cdot x_{j}}{\left|x_{i}||x_{j}\right|}$$

We fully utilize matrices constructed from spatial location data and gene expression. We compute the results obtained by separately training with different views (Fig. 7C). Subsequently, STMVGAE is tested for integrating clustering results obtained through different view training (Supplementary Tables S4, S5).

### 2.6 Multi-view variational graph auto-encoders network

Since STMVGAE adopts a multi-view model framework, it takes the enhanced gene expression to perform graph convolution operations on different views to extract view-specific potentially low-dimensional representations, we propose to learn a mapping function $f(A^{\left(i\right)},X,\theta (i))\to Z^{\left(i\right)}$, which maps the enhanced gene expression matrix *X* into a latent feature representation $Z^{\left(i\right)}$ based on the view $A^{\left(i\right)}$, with θ(i) being the model parameters.

In order to accomplish the spatial domain identification task, it is necessary to first incorporate multiple views into gene expression through STMVGAE. To achieve this goal, we utilize a fully connected layer to enhanced gene expression ($\overset{˜}{GE}$) to get $Z_{f}$, and then obtain low-dimensional latent embeddings *Z* through a two-layer graph convolutional neural network (GNN). Specifically, we employ GNNs in PyG to build our variational graph auto-encoders, and our model can choose different GNNs for training, including GCNConv, GATConv [37], SuperGATConv [38], SGConv [39], etc (Fig. 7A). We take $A^{\left(i\right)}$ and the enhanced gene expression $\overset{˜}{GE}$ as input to STMVGAE, and generate the graph embedding $Z^{\left(i\right)}$ as output. The GCN layer can generate low-dimensional embeddings, following Kipf and Welling [40], which is defined as:

(5) $$Z_{l}^{\left(i\right)}=GNN(Z_{f},A^{\left(i\right)})=\sigma (D^{(i)-\frac{1}{2}}{\overset{ˆ}{A}}^{\left(i\right)}D^{(i)-\frac{1}{2}}Z_{l-1}^{\left(i\right)}W_{l-1})$$

where *σ*(⋅) is an activation function such as ReLU (Rectified Linear Unit). ${\overset{ˆ}{A}}^{\left(i\right)}$ = $A^{\left(i\right)}$ + $I_{N}$, $A^{\left(i\right)}$ is the *i*-th adjacency matrix and $I_{N}$ is the identity matrix with dimension N×N, ${\overset{ˆ}{A}}^{\left(i\right)}$ is the *i*-th adjacency matrix with a self-loop. $D^{\left(i\right)}$ is the degree matrix corresponding to $A^{\left(i\right)}$. $W_{\left(l-1\right)}$ is the trainable weight matrix of the *l*-th layer, $Z_{f}=Z_{0}$, $Z_{\left(l\right)}$, $Z_{\left(l-1\right)}$ are the input and output of the *l*-th layer.

We design two-layer GNNs to learn low-dimensional representations. The first layer reduces the feature dimension and obtains $Z_{g}$, and the second layer yields *μ* and $log\sigma^{2}$, where *μ* is the mean of the low-dimensional representation, and $log\sigma^{2}$ is the variance of the low-dimensional representation, which is defined as:

(6) $$\mu =GNN_{\mu }(Z_{g},A)$$

(7) $$log\sigma^{2}=GNN_{\sigma }(Z_{g},A)$$

The learnable parameters of the first and second layers are $W_{1}$ and $W_{2}$, respectively. $GNN_{\mu }$ and $GNN_{\sigma }$ share $W_{1}$, but $W_{2}$ is different, we then use the reparameterization trick to obtain Z:

(8) $$Z=\mu +log\sigma^{2}\times \varepsilon$$

where ε∼N(0,1), specifically, *ε* is a random noise that follows a Gaussian distribution.

After obtaining the low-dimensional representation *Z*, we use a simple inner product decoder to reconstruct the adjacency matrix. The reconstructed adjacency matrix is as follows:

(9) $$P(A|Z)=\prod_{i=1}^{N}\prod_{j=1}^{N}p(A_{ij}|z_{i},z_{j})$$

(10) $$\overset{˜}{A}=p(A_{ij}=1|z_{i},z_{j})=sigmoid(ZZ^{T})$$

To calculate the probability that spot *i* and spot *j* are directly connected by an edge. $\overset{˜}{A}$ is the reconstructed adjacency matrix. We define the reconstructed adjacency matrix loss as:

(11) $$L_{A}=||A-\overset{˜}{A}|{|}^{2}$$

By minimizing the error between *A* and $\overset{˜}{A}$, more spatial location information can be retained to achieve better clustering performance.

In addition to reconstructing the adjacency matrix, we can also utilize a decoder to reconstruct the gene expression matrix *X*, preserving more content information by constraining the model as follows:

(12) $$L_{X}=||X-\overset{˜}{X}|{|}^{2}$$

where *X* is the enhanced gene expression as the raw input of STMVGAE, and $\overset{˜}{X}$ is the reconstructed input by the STMVGAE decoder. In this way, STMVGAE integrates both the spatial coordinate and the content of samples into a discriminative representation for clustering.

In addition to the loss functions $L_{A}$ and $L_{X}$ that we constructed through the above, following Kingma and Welling [41], we also consider the Kullback–Leibler divergence between the node representation vector distribution and the normal distribution, defined as:

(13) $$L_{KL}=E_{q(Z|X,A)}[log\;p(A|Z)]-KL[q(Z|X,A)||p(Z)]$$

where $E_{q(Z|X,A)}[log\;p(A|Z)]$ is the binary cross-entropy function, $p(Z)=\prod_{i}N(0,I)$.

### 2.7 Self-supervised module

We incorporate the Deep Embedded Clustering (DEC) [42] method into STMVGAE, combining this module with deep unsupervised clustering to optimize clustering performance during training through a self-supervised approach, denoted as the self-supervised module. Initially, we use a variational graph autoencoder (VGAE) to compress the augmented gene expression data into a low-dimensional latent representation *Z*. A clustering layer, denoted as ${\left\{\mu_{j}\right\}}_{j=1}^{J}$, is subsequently introduced within the encoder's latent space, where *J* represents the total number of clusters. During this pre-training phase, optimization of the self-supervised module is intentionally omitted. In the main training phase, Mclust [28] is employed to perform clustering on the *Z* representations, with the mean of samples in each identified cluster serving as the initial cluster centers. These centers act as the starting points for clustering. The clustering layer in the self-supervised module specifically stores these initial cluster centers, refining them through iterative optimization.

We use the Student's t-distribution similarity [10] to measure the similarity between each spot and the cluster centers. This similarity is then converted into the probability $q_{ij}$ that each spot belongs to a specific cluster, as given by the following equation:

$$q_{ij}=\frac{{\left(1+||z_{i}-\mu_{j}|{|}^{2}\right)}^{-1}}{\sum_{j^{\prime }}{\left(1+||z_{i}-\mu_{j^{\prime }}|{|}^{2}\right)}^{-1}}$$

where $z_{i}$ denotes the *i*-th spot in the low-dimensional representation *Z*, and $\mu_{j}$ represents the *j*-th cluster center. The value $q_{ij}$ calculates the soft assignment probability, indicating the likelihood that the *i*-th spot is assigned to the *j*-th cluster center.

Furthermore, the self-supervised module generates a target distribution in which high-confidence samples (i.e., spots that are closer to the cluster centers) are assigned higher weights. This target distribution is primarily constructed by emphasizing the peaks of the current soft assignment, enabling the model to more effectively distinguish between clusters. This target distribution is denoted as $p_{ij}$:

(14) $$p_{ij}=\frac{q_{ij}^{2}/\sum_{i}q_{ij}}{\sum_{j^{\prime }}(q_{ij_{\prime }}^{2}/\sum_{i}q_{ij^{\prime }})}$$

where $p_{ij}$ represents the probability that the *i*-th spot belongs to the *j*-th cluster in the target distribution, and $\sum_{i}q_{ij}$ is the sum of the assignment probabilities of all spots to cluster *j*, representing the normalized assignment for cluster *j*.

The self-supervised module then minimizes the Kullback-Leibler (KL) divergence between the target distribution $p_{ij}$ and the current soft distribution $q_{ij}$, adjusting both the cluster centers and the spot assignments to progressively refine the clustering results. In each iteration, the cluster centers shift gradually as the network parameters are updated, allowing them to better align with the underlying data distribution:

(15) $$L_{DEC}=KL(P|Q)=\sum_{i}\sum_{j}p_{ij}log\frac{p_{ij}}{q_{ij}}$$

### 2.8 Overall loss function

The overall objective loss function of STMVGAE can be summarized as:

(16) $$\begin{array}{c} L_{overall}=\lambda_{1}L_{A}+\lambda_{2}L_{X}+\\ \lambda_{3}L_{KL}+\lambda_{4}L_{DEC} \end{array}$$

where $\lambda_{1},\lambda_{2},\lambda_{3},\lambda_{4}$ are the hyper-parameters balancing the importance of difference losses. In the above loss function, three reconstruction losses and one self-supervised loss are included, which optimize the low-dimensional representation of the clustering tasks from different perspectives.

### 2.9 Consensus clustering of STMVGAE

This paper proposes a consensus clustering framework, STMVGAE, to solve the problem that single-view methods cannot fully capture neighbor information. STMVGAE captures the characteristics of the original data from different perspectives and obtains the most stable clustering results through a consensus clustering strategy. The steps of SpatialCVGAE are as follows:

**Step 1. Multi-view clustering:** We construct multiple spatial graphs and incorporate enhanced gene expressions as inputs to variational graph autoencoders (VGAEs). This step allows for the acquisition of spatial structures from various perspectives. By clustering the latent representations $Z^{\left(i\right)}$ learned through multi-view VGAEs, a variety of clustering outcomes $Y^{\left(1\right)},Y^{\left(2\right)},Y^{\left(3\right)},...,Y^{\left(N\right)}$ are generated.

**Step 2. Constructing the consensus matrix:** We calculate the clustering consensus matrix *C* by following this process:

(17) $$C_{ij}=\frac{\sum_{n=1}^{N}I(y_{i}^{\left(n\right)}=y_{j}^{\left(n\right)})}{N}$$

where *I*(⋅) is the indicator function, $y_{i}^{\left(m\right)}$ and $y_{j}^{\left(m\right)}$ indicate that they are spot *i* and spot *j* in the *n*-th clustering result $Y^{\left(n\right)}$. For each clustering result, $C_{ij}$ is a binary similarity matrix constructed based on the clustering result. If $y_{i}^{\left(m\right)}$ and $y_{j}^{\left(m\right)}$ are predicted to belong to the same spatial domain, the similarity is 1, otherwise it is 0. The consensus matrix is calculated by averaging all similarity matrices of individual clusterings. Consequently, we compute the connectivity matrix *C* for $Y^{\left(1\right)}$,$Y^{\left(2\right)}$,$Y^{\left(3\right)}$,...,$Y^{\left(M\right)}$.

**Step 3. Clustering of consensus matrix:** We use a consensus clustering strategy based on hierarchical clustering. This strategy analyzes the consensus matrix *C* to identify the most stable consensus clustering label $Y^{\left(⁎\right)}$. Hierarchical clustering can preserve the hierarchical relationship between samples and provide more comprehensive group structure information.

### 2.10 Baselines and evaluation metrics

We compare STMVGAE with several other state-of-the-art methods for identifying spatial domain tasks, including the non-spatial method SCANPY [35] and five spatial methods stLearn [14], SEDR [19], SpaGCN [17], DeepST [20] and STAGATE [18] (Supplementary Note 1.1).

We select four common unsupervised clustering evaluation indicators for quantitative comparison, which are adjusted rand index (ARI) [43], normalized mutual information (NMI), homogeneity score (HS), and Purity. The parameter settings of the comparison methods and the detailed calculation method of the unsupervised clustering evaluation matrix are provided in the Supplementary Note 1.2.

### 2.11 Downstream analysis

We demonstrated the ability of STMVGAE to perform downstream tasks on each dataset, including spatially variable gene (SVG) identification, UMAP visualization [11], PAGA trajectory inference [31], denoising, and batch integration. In our downstream task analysis, the denoising task utilized the reconstructed input generated from the Radius-balltree view alongside the original input. For other tasks, we relied on embeddings derived from the Radius-balltree view, as these embeddings exhibited notable stability and representativeness throughout our experiments. In the task of identifying spatially variable genes (SVGs), we improved upon the SpaGCN method [17]. Specifically, by integrating the gene expression features of the target cluster and its neighboring clusters, we employed a spatially neighborhood−based differential expression analysis to identify genes with significant spatial specificity.

## 3 Results

### 3.1 Overview of experimental results

STMVGAE achieved competitive results in spatial domain identification for all datasets, demonstrating its excellent generalization ability (Table 2). Moreover, besides the spatial domain identification task, STMVGAE could also perform a variety of downstream tasks. We demonstrated the capabilities of STMVGAE in UMAP visualization, PAGA trajectory inference, spatially variable genes (SVGs) identification, denoising, and batch integration (Fig. 1). The specific experimental settings are available at Supplementary Note 1.3.

**Table 2:** Clustering results of STMVGAE and baseline methods on all datasets. The best results are in bold black.

Methods	DLPFC				BCDC				Melanoma				BRCA			
	ARI	NMI	HS	Purity	ARI	NMI	HS	Purity	ARI	NMI	HS	Purity	ARI	NMI	HS	Purity
stLearn [14]	0.356	0.526	0.533	0.646	0.501	0.456	0.425	0.855	-	-	-	-	0.588	0.653	0.643	0.646
SEDR [19]	0.499	0.643	0.631	0.725	0.187	0.077	0.083	0.799	0.281	0.389	0.418	0.754	0.485	0.660	0.644	0.566
SpaGCN [17]	0.411	0.543	0.520	0.614	0.334	0.378	0.348	0.789	0.415	0.447	0.423	0.694	0.441	0.626	0.619	0.552
DeepST [20]	0.476	0.620	0.606	0.689	0.459	0.389	0.354	0.838	-	-	-	-	0.546	0.680	0.663	0.642
STAGATE [18]	0.501	0.645	0.624	0.727	0.442	0.337	0.319	0.836	0.410	0.438	0.418	0.719	0.460	0.688	0.666	0.561
STAMARKER [23]	0.527	**0.662**	0.640	0.735	0.450	0.358	0.337	0.838	0.429	0.442	0.427	0.740	0.461	0.689	0.667	0.562
**STMVGAE(ours)**	**0.562**	0.638	**0.648**	**0.789**	**0.730**	**0.584**	**0.583**	**0.931**	**0.480**	**0.468**	**0.480**	**0.804**	**0.660**	**0.699**	**0.689**	**0.678**

### 3.2 STMVGAE can accurately identify the layers on the DLPFC dataset

Firstly, we used the DLPFC dataset from 10x Genomics [3] to test the performance of the STMVGAE spatial domain identification.

**Fig. 2:**
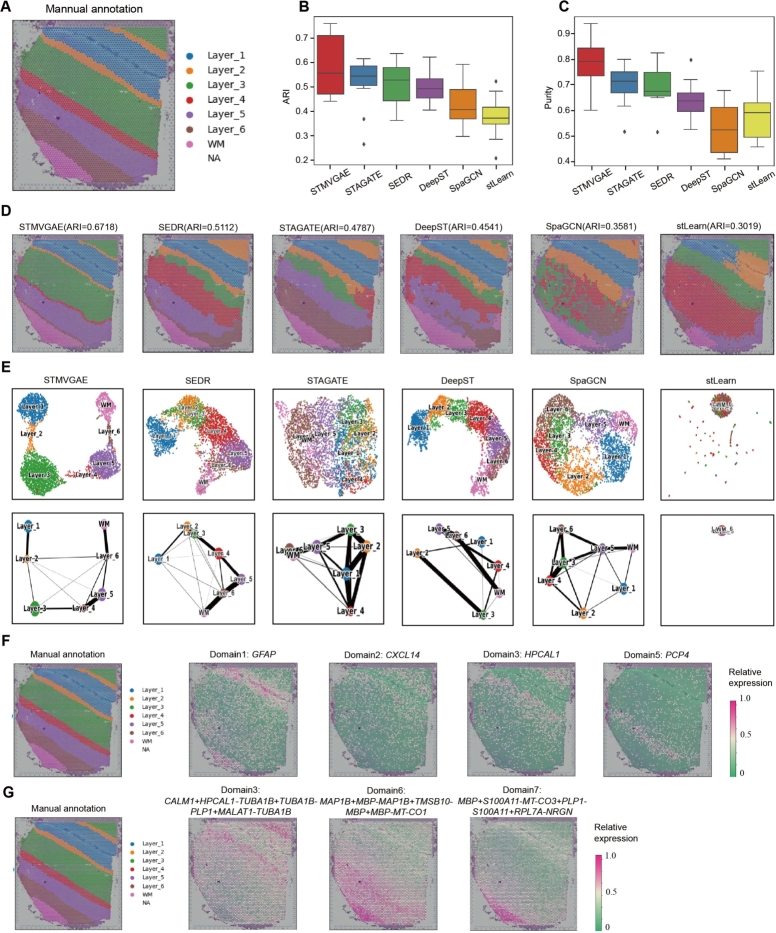
STMVGAE can accurately identify spatial domains and SVGs on the DLPFC dataset. (**A**) Manually annotated hierarchies for 151508 slice in the DLPFC dataset. (**B**) Box plots of STMVGAE and five baseline methods on 12 slices with ARI. (**C**) Box plots of STMVGAE and five baseline methods on 12 slices with Purity. (**D**) Domain identification on 151508 slice by STMVGAE, SEDR, STAGATE, DeepST, SpaGCN, and stLearn. (**E**) UMAP visualization and PAGA trajectory on 151508 slice by STMVGAE, SEDR, STAGATE, DeepST, SpaGCN, and stLearn. (**F**) Spatial expression patterns of SVGs detected by STMVGAE in 151508 slice. (**G**) Spatial expression patterns of metagenes detected by STMVGAE on 151508 slice.

We selected slice 151508 from the DLPFC for display (Fig. 2). To facilitate comparison with other methods, we manually reordered the spatial domain identification results generated by stLearn, SpaGCN, DeepST, SEDR, and STAGATE according to the cortical layer structure sequence labeled in Fig. 2A. As shown in Fig. 2D and Supplementary Fig. S1, stLearn exhibited the poorest performance, incorrectly assigning Layer_1 and Layer_2 together. SpaGCN correctly identified Layer_1 and the white matter layer (WM), but the spots in other layers were mixed. DeepST displayed a clearer hierarchical structure, but encountered difficulty in allocating spots between multiple layers. In contrast, STAGATE and SEDR showed relatively clear boundaries between layers, but STAGATE misplaced Layer_1, and SEDR exhibited an unsmooth boundary between Layer_5 and Layer_6. STMVGAE demonstrated superior performance among these methods, with clear boundaries between layers and precise spot allocation. Notably, STMVGAE exhibited exceptional accuracy in identifying narrow areas such as Layer_2. In Fig. 2B, C, STMVGAE achieved the highest ARI value of 0.67 for slice 151508, surpassing the other methods, all of which scored below 0.6. Additionally, STMVGAE attained the highest Purity value.

STMVGAE proved effective for both UMAP visualization and PAGA trajectory analysis. We visualized the low-dimensional embeddings and presented the spatial trajectories (Fig. 2E and Supplementary Fig. S2). Taking the UMAP visualization analysis of 151508 slices as an example, stLearn did not make full use of the spatial coordinate information, and the UMAP visualization could not assign each class to a reasonable spatial location. There was no obvious boundary between different layers of STAGATE and SpaGCN, different spatial domains of STAGATE were almost squeezed together and SpaGCN formed a ring structure. DeepST and SEDR achieved relatively good results, but the PAGA trajectory of DeepST was disordered, and the layer_2 and layer_3 of SEDR were mixed. In contrast, STMVGAE presented spots that clearly organized the different layers and accurately reflected the developmental sequence of the cortical layer [44], not only did STMVGAE clearly organize the boundaries between each layer, but the spots of the different layers were not located in the other layers.

To further explore downstream tasks, we used the same procedure as SpaGCN [17] to identify SVGs (Fig. 2F). We detected a total of 35 SVGs on the 151508 slice, which were dispersed across different domains. These included 4 SVGs in domain 0, 8 SVGs in domain 2, 1 SVG in domain 3, 21 SVGs in domain 4, and 1 SVG in domain 6. We utilized different colors to represent the relative expression levels of related genes, and the different domain SVGs identified by STMVGAE in Fig. 2F matched the artificially annotated cortical layer structure in Fig. 2A.

Since some neuronal layers were difficult to label with a single gene, we constructed metagenes to label specific domains (Fig. 2G). Due to the fewer number of spots in Layer_2, it was difficult to detect genes enriched in this domain. Therefore, we significantly enhanced the expression pattern by increasing genes such as *CXCL14*, *HPCAL1*, *MBP*, etc.

### 3.3 STMVGAE denoises gene expressions for better characterizing spatial expression patterns

Raw spatial transcriptomics data were limited by high noise and high dropout events, which could interfere with the accuracy of gene expression analysis. Therefore, a reliable approach should have been able to separate irrelevant noise from the raw data while preserving critical organizational information.

**Fig. 3:**
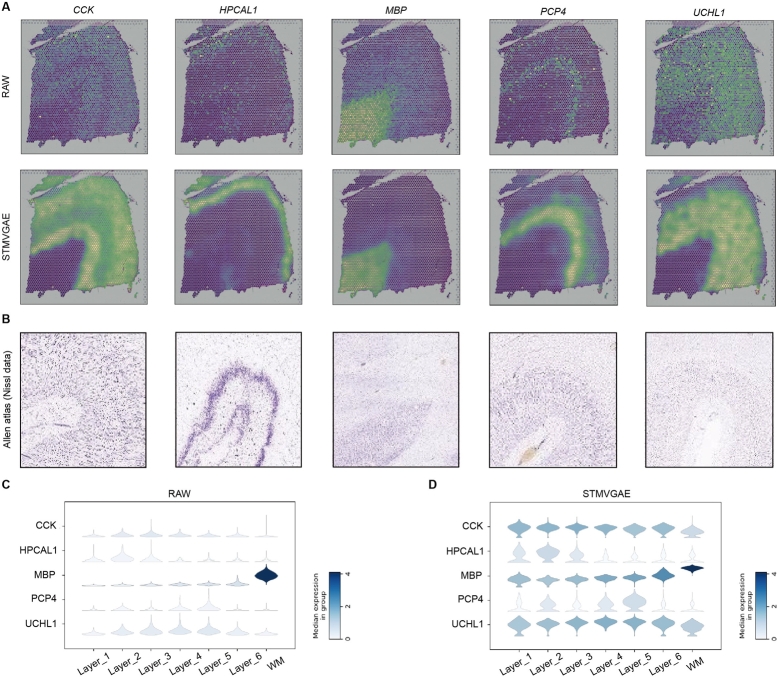
STMVGAE enhances the spatial patterns of layer-marker genes in the DLPFC dataset. (**A**) Visualizations of the raw spatial expressions and STMVGAE denoised ones of six layer-marker genes in the DLPFC section 151676. (**B**) Nissal images of the adult human brain from the Allen Human Brain Atlas. (**C**) The violin plot of cortical marker gene expression imputed by STMVGAE.

STMVGAE could denoise and impute gene expressions. We employed STMVGAE to reduce noise in the DLPFC dataset to better visualize the spatial pattern of genes. As shown in Fig. 3A, we compared the expression of five raw layer-marker genes (*CCK, HPCAL1, MBP, PCP4, UCHL1*) from slice 151676 of DLPFC with STMVGAE reconstructed expression. In the raw data, we found that there was a strong confounding effect on the five marker genes, and the data points for high expression of this gene were scattered. We used the reconstituted data from STMVGAE to accurately describe the boundaries of each marker gene. The results showed that STMVGAE could effectively capture the global probability distribution of ST data and reconstruct the original data. We validated the laminar enrichment shown by STMVGAE against publicly available in situ hybridization (ISH) data from the Allen Human Brain Atlas [45] (Fig. 3B). In addition, we compared the original expression with the denoising expression using a violin plot (Fig. 3C,D) and found that the STMVGAE-enhanced data were more consistent with the manual tissue structure annotation, significantly enhancing the spatial pattern of hierarchical marker genes. The experimental results indicated that STMVGAE was an effective method for imputing gene expression, further validating the superior performance of our method.

### 3.4 STMVGAE can accurately identify spatial domains on the BRCA dataset

Next, we analyzed the human breast cancer (BRCA) dataset from the 10x Genomics [3] platform, the data mainly included four types of spots: ductal carcinoma in situ/lobular carcinoma in situ (DCIS/LCIS), healthy tissue (Healthy), invasive ductal carcinoma (IDC), and tumor surrounding regions with low features of malignancy (Tumor edge).

**Fig. 4:**
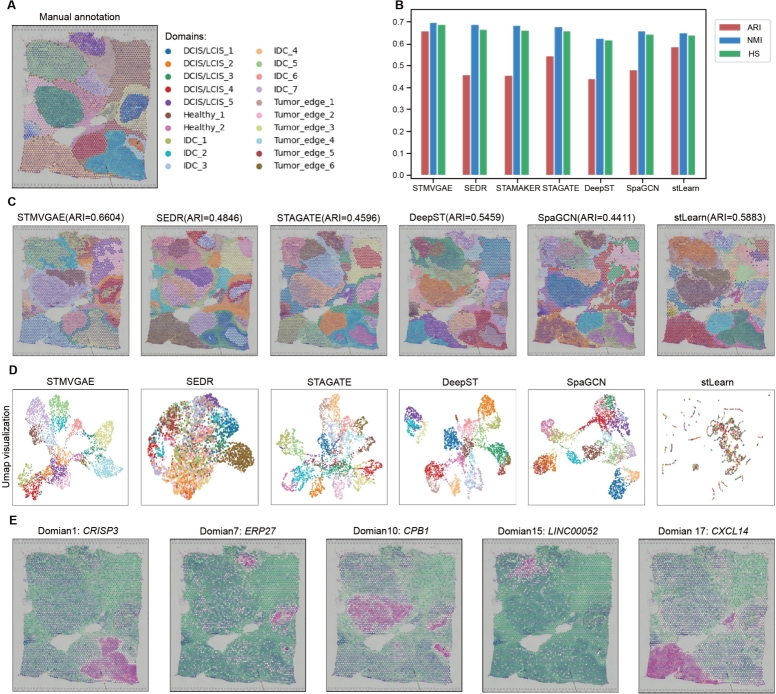
STMVGAE can accurately identify spatial domains and SVGs on the BRCA dataset. (**A**) Manual annotating of BRCA dataset based on pathological features. (**B**) Bar plots of STMVGAE and five baseline methods on the BRCA dataset with three different evaluation matrices. (**C**) Domain identification on the BRCA dataset by STMVGAE, SEDR, STAGATE, DeepST, SpaGCN, and stLearn. (**D**) UMAP visualization and PAGA trajectory on the BRCA dataset by STMVGAE, SEDR, STAGATE, DeepST, SpaGCN, and stLearn. (**E**) STMVGAE Spatial expression patterns of SVG detected on different spatial domains in the BRCA dataset.

As shown in Fig. 4, we presented the results of six deep learning methods, namely STMVGAE, SEDR, STAGATE, DeepST, SpaGCN, and stLearn, for spatial domain identification on the BRCA dataset. Among the six methods, SpaGCN performed the worst in the spatial domain identification tasks, exhibiting chaotic spot allocation in multiple spatial domains such as IDC_2 and IDC_4. STAGATE, SEDR, and DeepST showed spot allocation confusion and unsmooth boundaries, and they were unable to accurately identify the IDC_2 area. stLearn exhibited some outliers in different spatial domains. In contrast, the results of STMVGAE displayed clearer boundaries and more reasonable spot allocation. STMVGAE demonstrated better identification of IDC_2, IDC_4, DCIS/LCIS_4, and other areas (Fig. 4A,C). The ARI, NMI, and HS values of STMVGAE were the highest among all methods, with the ARI value of 0.66, while other comparison methods scored lower than 0.6 (Fig. 4B).

Fig. 4D showed the results of UMAP visualization of the low-dimensional embeddings of the six methods. There was a mess in stLearn. In the visualizations of SpaGCN and DeepST, there was a discontinuity of spatial domains, and some spatial domains existed separately from the whole. There were no very clear boundaries between the SEDR space domains. STAGATE appeared to have a slight blend of spots between different domains. The UMAP visualization of STMVGAE revealed that most of the spots were well organized and had clear boundaries between different domains. With the identified domains, we further identified the SVGs in different spatial domains. We detected a total of 468 SVGs on the BRCA dataset, which were dispersed across different domains. As shown in Fig. 4E, in the task of identifying SVGs, STMVGAE accurately identified the *CXCL14* in domain 17, which had been proven to have prognostic significance in breast cancer [46].

### 3.5 STMVGAE can accurately distinguish tumor areas and non-tumor areas on the BCDC dataset

**Fig. 5:**
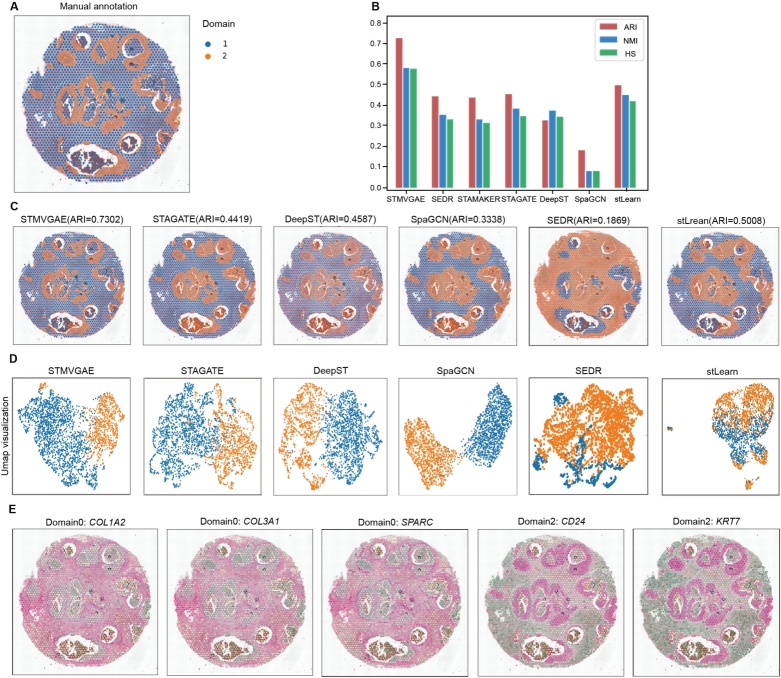
STMVGAE can accurately distinguish tumor areas and non-tumor areas on the BDCD dataset. (**A**) Manual annotating of BCDC dataset based on pathological features. (**B**) Bar plots of STMVGAE and five baseline methods on the BCDC dataset with three different evaluation matrices. (**C**) Domain identification on the BCDC dataset by STMVGAE, SEDR, STAGATE, DeepST, SpaGCN, and stLearn. (**D**) UMAP visualization and PAGA trajectory on the BCDC dataset by STMVGAE, SEDR, STAGATE, DeepST, SpaGCN, and stLearn. (**E**) STMVGAE Spatial expression patterns of SVG detected on different spatial domains in the BCDC dataset.

We applied STMVGAE to analyze the human breast cancer ductal carcinoma (BCDC) dataset. The dataset had been manually annotated with two regions: Domain 1 for non-tumor regions and Domain2 for tumor regions. Since there were only two categories in the BCDC dataset, the identification of tumor regions and non-tumor regions was the main task of spatial domain identification in the BCDC dataset. As indicated by manual annotation in Fig. 5A, a larger proportion of non-tumor regions were present in the BCDC dataset. SEDR achieved the worst results, incorrectly identifying more healthy areas in the dataset as tumor areas. The spatial domain recognition results obtained by the SpaGCN and stLearn failed to successfully divide the tumor region and the non-tumor regions at the central location. Although the spatial domain results generated by DeepST and STAGATE successfully identified the tumor region and non-tumor region in the middle position, the boundary of the region division was not clear, and the division ratio within the region was incorrect. STMVGAE achieved good results on BCDC dataset, and it was the closest to manual annotation for the identification of intermediate regions, and it was also very accurate for the identification of peripheral tumor regions (Fig. 5C, D). At the same time, STMVGAE also achieved the highest ARI value in all evaluation indicators on the dataset, among which the value of ARI was 0.73, and all other comparison methods except stLearn were lower than 0.5 (Fig. 5B).

To further explore the spatial domains we generated, we performed SVG identification on the BCDC dataset. A total of 1364 SVGs were identified on the BCDC dataset, including 78 SVGs in non-tumor region 1 and 1286 SVGs in tumor region 2. We speculated that there were only two types of BCDC datasets, so there were more SVGs that could be identified by STMVGAE on BCDC dataset (Fig. 5E).

### 3.6 STMVGAE can accurately identify spatial domains on the melanoma dataset

We evaluated the performance of STMVGAE on melanoma cancer from Thrane et al. [34]. There were three distinct areas in this data: melanoma, stroma, and lymphoid tissue, with an additional unannotated area [47]. We also used four evaluation indicators to measure the performance of STMVGAE in this dataset. The simplified version of STMVGAE performed better than several other comparison methods on this dataset, and STMVGAE achieved significant improvements. STMVGAE attained the highest ARI value of 0.48 among all competing methods, and it was the only one among several comparison methods with a Purity value of more than 0.8 (Table 2).

### 3.7 STMVGAE is capable of analyzing high-resolution olfactory dataset

**Fig. 6:**
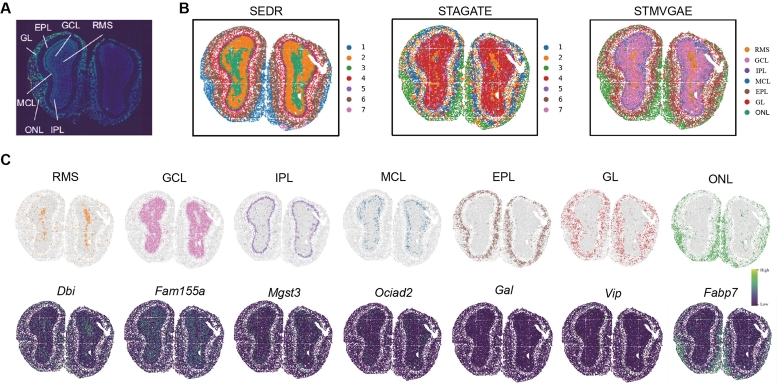
STMVGAE is able to accurately identify laminar structures in mouse olfactory bulb datasets. (**A**) Laminar organization of mouse olfactory bulb annotated in the DAPI-stained image generated by Stereo-seq. (**B**) Spatial domains generated by SEDR, STAGATE, and STMVGAE embeddings in the Stereo-seq mouse olfactory bulb tissue section. (**C**) Visualization of spatial domains identified by STMVGAE and the corresponding marker genes.

To further test STMVGAE spatial domain identification, we also tested it on a mouse olfactory bulb cell dataset from the high-resolution Stereo-seq [6] platform, whose sequencing technology could achieve submicrometer and subcellular resolution. The laminar structure of the data was divided from inside to outside: the rostral migratory stream (RMS), the granule cell layer (GCL), the internal plexiform layer (IPL), the mitral cell layer (MCL), the glomerular layer (GL), and the olfactory nerve layer (ONL). We chose two methods for comparison, SEDR and STAGATE. The performance of these two methods was second only to STMVGAE on the DLPFC dataset, as shown in Fig. 6B. STMVGAE could well identify the laminar flow structure of mouse olfactory bulb cell data, and it was consistent with the artificially annotated laminar flow structure [19] (Fig. 6A). The SEDR method was not accurate enough in identifying the rostral migratory stream (RMS) spatial domain. Not only did it not correctly identify the range of this spatial domain, but it also had no clear boundary with the granule cell layer (GCL). The STAGATE method was also not accurate enough in identifying the rostral migratory stream (RMS) spatial domain. It did not identify the spatial domain as a continuous area, and STAGATE was not accurate enough in the outer spatial domain internal plexiform layer (IPL), granule cell layer (MCL), and glomerular layer (GL) spot allocation, which was confusing. In contrast, STMVGAE accurately identified the rostral migratory stream (RMS), and for the granule cell layer (GCL), internal plexiform layer (IPL), granule cell layer (MCL), glomerular layer (GL), and the olfactory The recognition of nerve layer (ONL) was more accurate (Fig. 6C). We verified our results by detecting marker genes in each layer, and the results showed that Dbi and Fam155a were strongly expressed on the rostral migratory stream (RMS) and the granule cell layer (GCL). Our experimental results were consistent with some previous studies [48], [49]. The above experimental results showed that STMVGAE could process ST data at different spatial resolutions.

### 3.8 STMVGAE corrects for batch effects

In recent years, the application of spatial transcriptome sequencing technology greatly broadened people's horizons, enabling them to gain insight into the diversity of cell composition and gene expression status in tissues. However, different protocols and techniques between different spatial transcriptomics data complicated the integration of the data. As with scRNA-seq data, removing batch effects from spatial transcriptomics data was a significant challenge. In this section, we tested the joint embedding of multi-batch data with different expression patterns in the DLPFC dataset. We projected the data into a latent space using STMVGAE and two other comparison methods, and then performed batch effect correction in the latent space using Harmony [50]. The experiments demonstrated that the integration of STMVGAE with the Harmony tool effectively reduced batch effects, outperforming the comparison methods.

**Fig. 7:**
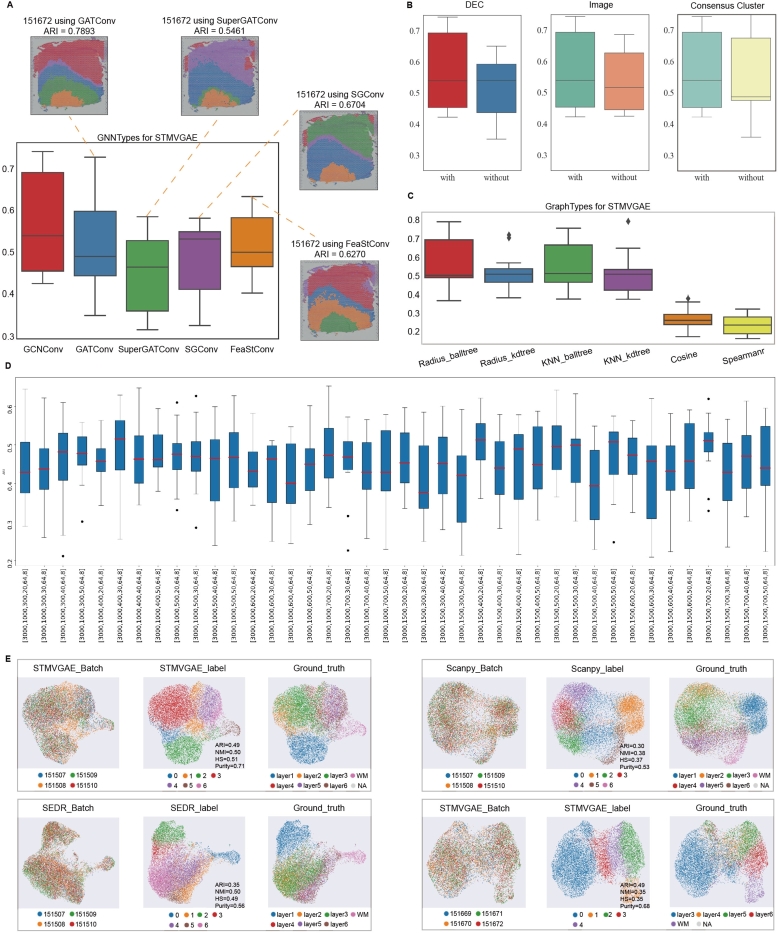
Ablation study and STMVGAE multi-slice joint analysis. (**A**) The ARI pirate graph of five GNN types, each of which was evaluated on 12 DLPFC slides, respectively. Spatial domain distributions of slides 151672 with various networks (GATConv, SuperGATConv, SGConv and FeaStConv) are displayed, respectively. (**B**) ARI box plots showing low-dimensional representation learning in STMVGAE with or without DEC self-supervised module, spatial data augmentation, and consensus clustering. (**C**) The ARI boxplot compares six methods for constructing adjacency matrices in STMVGAE. (**D**) The clustering accuracy of simple-STMVGAE with different hyperparameters in all 12 sections. The hyperparameters are selected by grid search of the first three linear layers and the last two graph convolutional layers of STMVGAE. (**E**) UMAP plots of multi-slice joint analysis. They represent batches, recognition spatial domains, and ground truth labels, respectively.

We compared the performance of STMVGAE, SCANPY, and SEDR in batch data integration processing using the deep method SEDR versus the non-deep method SCANPY (Fig. 7E and Supplementary Fig. S3). For batch integration, we used Harmony technology [50], which demonstrated superior performance in scRNA-seq. We selected the first four slices in the DLPFC dataset for integration (151507, 151508, 151509, 151510), and the visualization obtained by SCANPY was heavily mixed with speckles between different domains. For STMVGAE and SEDR embedding, cells in different cortical layers exhibited a distinct order of separation and development, while SEDR did not identify small areas as clearly as STMVGAE. We also obtained the manual annotation of the joint batch data by integrating the manual annotation of each slice. We calculated the results obtained by three different methods and the manual annotation of four indicators, the NMI values obtained by STMVGAE and SEDR were comparable, and the other three indicators were the highest values obtained by STMVGAE, and the ARI value of STMVGAE was 0.49 (Supplementary Fig. S3A). Considering that the data in the DLPFC dataset presented different data patterns, we divided the DLPFC dataset according to the data schema for joint batch analysis. In addition, we also performed a joint batch analysis of two other slices with different data patterns on the DLPFC dataset using STMVGAE. We used STMVGAE to perform a combined batch analysis of the middle four slices (151669, 151670, 151671, 151672) and the last four slices (151673, 151674, 151675, 151676). In the data analysis results of these two joint batches, it could be found that the UMAP visualization results obtained by STMVGAE could well isolate cortical layer cells with a clear developmental sequence (Supplementary Fig. S3B,C).

The ensemble results suggested that different methods embedding the contained spatial information might produce different results. The embedding obtained by STMVGAE could not only perform batch integration tasks on datasets with different expression patterns but also achieve the best performance compared to other methods. In conclusion, STMVGAE could effectively realize batch integration of spatial transcriptomics data with the help of the Harmony tool.

### 3.9 Ablation studies

We systematically evaluated STMVGAE using the DLPFC dataset. First, we selected five different graph convolutional layers (GCNConv, GATConv, SuperGATConv, SGConv, FeaStConv) to calculate the ARI values for each of the 12 slices on the DLPFC dataset (Fig. 7A). GCN achieved good performance. Simultaneously, we tested the performance of different graph convolutional layers on the 151672 slices and obtained different hierarchical distributions under different network architectures. We found that STMVGAE achieved very good results on the 151672 tiles using different convolutional layers, and the ARI values were high. The self-supervised module indicated the goal regarding cluster optimization during training, which we initiated with or without the DEC self-supervised module. Secondly, the use of multi-modal data is a major feature of STMVGAE, and we tested the results with or without the integration of histological images features. Finally, we tested whether the results obtained by integrating different adjacency matrices using consensus clustering (Fig. 7B and Table 3).

**Table 3:** Clustering results of ablation experiments on all datasets. STMVGAE-w/o-ALL represents a model with only VGAE structure. STMVGAE-w/o-D, STMVGAE-w/o-I, and STMVGAE-w/o-C, respectively indicate whether the DEC self-supervision module, histological images information and consensus clustering are used. The best results are in bold black.

Methods	DLPFC				BCDC				Melanoma				BRCA			
	ARI	NMI	HS	Purity	ARI	NMI	HS	Purity	ARI	NMI	HS	Purity	ARI	NMI	HS	Purity
STMVGAE-w/o-ALL	0.517	0.619	0.629	0.740	0.533	0.486	0.454	0.867	0.456	0.463	0.446	0.701	0.594	0.684	0.670	0.656
STMVGAE-w/o-D	0.530	0.622	0.618	0.746	0.534	0.499	0.472	0.869	0.474	0.457	0.454	0.722	0.616	0.690	0.666	0.669
STMVGAE-w/o-I	0.540	0.626	0.618	0.747	0.447	0.348	0.332	0.857	-	-	-	-	0.609	0.681	0.672	0.634
STMVGAE-w/o-C	0.540	0.631	0.644	0.776	0.718	0.581	0.572	0.925	**0.480**	**0.468**	**0.480**	**0.804**	0.650	0.687	0.672	0.676
**STMVGAE(ours)**	**0.562**	0.638	**0.648**	**0.789**	**0.730**	**0.584**	**0.583**	**0.931**	-	-	-	-	**0.660**	**0.699**	**0.689**	**0.678**

Additionally, it is a major feature of STMVGAE to use different adjacency matrix construction methods to learn different data patterns. Therefore, we not only used spatial coordinate information but also gene expression to construct adjacency matrices. We also evaluated the impact of multiple adjacency matrix construction methods on STMVGAE performance (Fig. 7C). It was observed that the performance of constructing adjacency matrices using spatial coordinate location information significantly improved compared to that using gene expression, and the difference between the four adjacency matrices constructed using spatial coordinate location information was not significant. In order to find suitable model parameters, we conducted a grid search on the basic STMVGAE (without using multi-modal data, DEC self-supervised module, or consensus clustering). Our model used 3000 highly variable genes as input. Considering the retention of more information, the hyperparameters of the first linear layer were set to (1500,1000), the second linear layer to (700,600,500,400,300), and the third linear layer to (50,40,30,20). The hyperparameters of the graph convolutional layer were set to 64 and 8 (Fig. 7D).

### 3.10 The results of adjacency matrix integration with different similarities

To comprehensively evaluate the adjacency matrices constructed by different similarity measures as input to train STMVGAE, and subsequently use the final results obtained by consensus clustering integration, we introduced four adjacency matrix constructions: Radius_balltree, Radius_kdtree, KNN_balltree and KNN_kdtree, denoted as $A^{\left(1\right)}$, $A^{\left(2\right)}$, $A^{\left(3\right)}$, and $A^{\left(4\right)}$, respectively. The adjacency matrices constructed under four different similarity measures were used to train STMVGAE separately, and the results were plotted as box plots, showing their individual performance in Fig. 7C. It was observed that the adjacency matrices constructed with different similarity measures did not change significantly when used to train STMVGAE alone, indicating the robustness of STMVGAE.

We conducted experiments using any two of the four adjacency matrices as inputs to the model and calculated the ARI and NMI values on 12 slices of the DLPFC dataset. The results are shown in Supplementary Table S4 and Supplementary Table S5. Additionally, experiments were conducted to select any three of the four adjacency matrices as inputs, and all four adjacency matrices as inputs, followed by consensus clustering integration.

To better discuss the results, we saved the ARI values of 12 slices from the DLPFC dataset under each specific view combination and visualized them using boxplots with significance markers. The significance calculations were performed using the Wilcoxon rank sum test [51]. In the two-view combinations, $A^{\left(1\right)}+A^{\left(3\right)}$ performed the best. Therefore, we selected $A^{\left(1\right)}+A^{\left(3\right)}$ as the baseline and conducted a significance comparison with other combinations in the same group, namely $A^{\left(1\right)}+A^{\left(2\right)}$ and $A^{\left(2\right)}+A^{\left(4\right)}$. This analysis aims to confirm whether $A^{\left(1\right)}+A^{\left(3\right)}$ is significantly superior to other two-view combinations, highlighting its advantages in information representation and feature capturing.

**Fig. 8:**
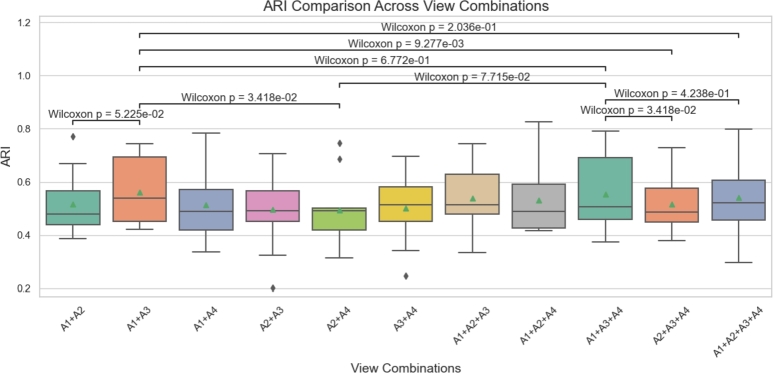
Box plot and significance markers of the ARI values of the STMVGAE method under multiple view combinations. The significance markers are calculated by the Wilcoxon rank sum test.

To further analyze whether increasing the number of views can improve performance, we compared the best two-view combination $A^{\left(1\right)}+A^{\left(3\right)}$ with two three-view combinations: the best-performing $A^{\left(1\right)}+A^{\left(3\right)}+A^{\left(4\right)}$ and the second-best $A^{\left(2\right)}+A^{\left(3\right)}+A^{\left(4\right)}$. We found that adding one view generally enhances clustering accuracy compared to two-view combinations. However, excessive integration of views (e.g., $A^{\left(1\right)}+A^{\left(2\right)}+A^{\left(3\right)}+A^{\left(4\right)}$) does not yield proportional benefits and may introduce noise, resulting in a slight decline in ARI performance. Meanwhile, NMI, HS and Purity values remained stable but showed no significant changes (Fig. 8 and Supplementary Figs. S4-S6).

Statistical analysis using the Wilcoxon test further validated these observations. Notably, the comparison between $A^{\left(1\right)}+A^{\left(2\right)}+A^{\left(3\right)}$ and $A^{\left(1\right)}+A^{\left(4\right)}$ revealed significant differences (*p* < 0.01), emphasizing the advantages of optimizing view selection. In contrast, no significant difference was observed between $A^{\left(1\right)}+A^{\left(2\right)}+A^{\left(3\right)}+A^{\left(4\right)}$ and $A^{\left(1\right)}+A^{\left(2\right)}+A^{\left(3\right)}$ (*p* > 0.05), indicating that when all views are included, performance improvements stabilize.

In conclusion, the results of our experiments indicate that increasing the number of views within a certain range significantly enhances performance. Multi-view configurations consistently outperform single-view setups, as evidenced by the comparison between Fig. 7C and Fig. 8. Notably, the median ARI for single-view configurations is concentrated around 0.5, whereas multi-view configurations demonstrate consistently higher values, underscoring their superior performance. $A^{\left(1\right)}+A^{\left(3\right)}$ serves as a suitable baseline combination. When aiming to enhance performance, $A^{\left(4\right)}$ can be prioritized to construct the three-view combination $A^{\left(1\right)}+A^{\left(3\right)}+A^{\left(4\right)}$. While adding views can indeed improve performance, there is a diminishing marginal return. The most notable improvement occurs when expanding from two views to three, whereas the performance gains are limited when moving from three views to four. This trend likely reflects the increasing redundancy of information in high-dimensional view combinations.

### 3.11 Verifying the effectiveness of STMVGAE learned embeddings

In spatial domain identification tasks within spatial transcriptomics, advanced methods predominantly employ either Mclust [28] or Louvain [9] clustering, both of which have achieved remarkable performance in this area. Notably, Mclust has emerged as the preferred choice due to its soft clustering capability, adaptability to various data distributions, and consistent stability. To discern whether the exceptional performance of STMVGAE stems primarily from Mclust's clustering efficiency or from STMVGAE's capacity to produce representative embeddings, we performed a comprehensive analysis.

**Fig. 9:**
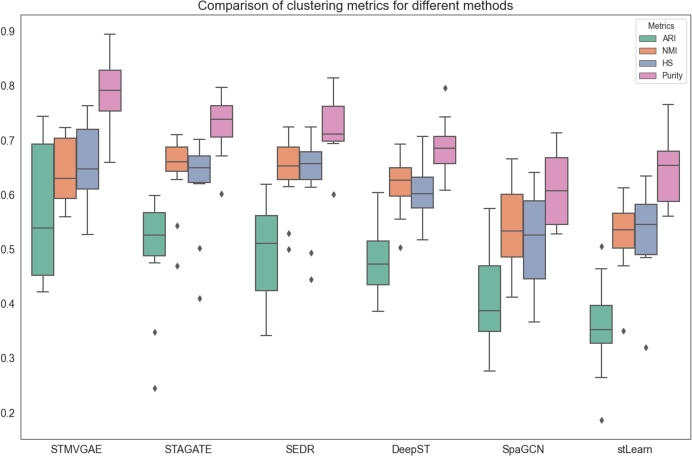
The performance of STMVGAE and other comparison methods on clustering metrics was evaluated using multiple boxplots.

**Fig. 10:**
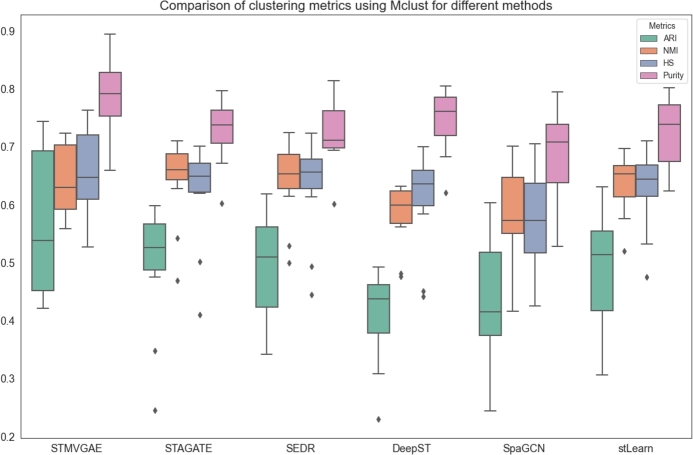
The performance of STMVGAE and other comparison methods on clustering metrics was evaluated using multiple boxplots, with all methods employing Mclust for clustering.

To test our hypothesis, we conducted two sets of experiments. In the first set, each method applied its native clustering approach (Fig. 9), while in the second set, all methods were standardized to use Mclust for clustering (Fig. 10). Among the baseline methods, STAGATE and SEDE inherently utilized Mclust clustering, whereas DeepST, SpaGCN, and stLearn relied on alternative clustering techniques. By analyzing the results of these experiments, we aimed to thoroughly evaluate the performance of STMVGAE under different clustering strategies and gain a deeper understanding of the factors that contributed to its superior performance.

As illustrated in Fig. 9, STMVGAE demonstrated notable advantages over other methods across various evaluation metrics, including ARI, HS, and Purity. This suggested that, under its native clustering configuration, the features (embeddings) learned by STMVGAE exhibited strong representational power, effectively capturing the intrinsic structures and information patterns within spatial transcriptomics data. Consequently, its native clustering approach produced relatively superior clustering outcomes. These findings indicated that STMVGAE effectively extracted critical information during the feature learning phase, enhancing its competitiveness across different clustering mechanisms.

As shown in Fig. 10, when Mclust clustering was applied to all methods, STMVGAE continued to maintain a significant lead in ARI and Purity metrics. Its HS performance also surpassed all other methods, while its NMI was comparable to that of STAGATE and SEDR. These results further demonstrated that the features learned by STMVGAE were both highly adaptable and effective. STMVGAE achieved the best performance under both clustering scenarios, primarily due to its numerous advantages in processing spatial transcriptomics data. By enhancing gene expression through image augmentation to mitigate data sparsity, mining spatial information from multiple views, and employing consensus clustering to integrate diverse clustering results, STMVGAE was able to generate high-quality, versatile features that effectively supported the clustering process across different clustering strategies. These findings not only highlighted the effectiveness of the STMVGAE framework but also established it as a reliable and high-performance solution for spatial domain identification tasks in spatial transcriptomics.

From Fig. 9 and Fig. 10, we observed several notable findings. The stLearn method demonstrated significant improvement, SpaGCN showed moderate improvement, while DeepST experienced a decline in performance. We speculated that these results might be due to the compatibility between the embeddings produced by each method and the Mclust clustering approach. Specifically, stLearn appeared to benefit greatly from Mclust, possibly because the distribution of its embeddings aligned well with the assumptions of Mclust. SpaGCN exhibited a smaller improvement, which could be attributed to its features being more locally spatially dependent. Conversely, the decline in the performance of DeepST suggested that the features generated by this method were better suited to alternative clustering strategies, such as the default Louvain clustering used by DeepST. These observations highlighted that applying Mclust universally to all methods was not always suitable and could lead to significant performance drops, as seen with DeepST. Achieving optimal results required aligning the generation of embeddings with the clustering strategies based on task requirements, thereby optimizing the compatibility between the two and improving overall performance.

## 4 Discussion and conclusion

With the rapid advancement of spatial transcriptomics, accurate identification of spatial domains is crucial for understanding tissue properties and cell functions. However, most existing methods do not fully leverage the diverse data types provided by spatial transcriptomics, including spatial location information and histological images.

In our study, we capitalize on the wealth of data offered by spatial transcriptomics, integrating gene expression, spatial location information, and histological images. We propose an unsupervised multi-view variational graph autoencoder, called STMVGAE, designed to learn low-dimensional representations. STMVGAE initially extracts information from histological images using a pre-trained convolutional neural network, which is then fused with preprocessed gene expression data to produce an enhanced input. Subsequently, STMVGAE trains enhanced gene expression and multi-view data separately using graph convolution to obtain multiple view-specific low-dimensional latent embedding representations, which are utilized for downstream tasks. Additionally, STMVGAE employs Mclust clustering to cluster these latent embedding representations, obtaining the distribution of spots in each spatial domain. Finally, we use consensus clustering to integrate the spot distribution results in the spatial domain for final spot prediction, thereby facilitating spatial domain identification. STMVGAE utilizes a enhanced multi-view approach to construct multiple loss functions for training, enhancing model performance. The integration of clustering results through consensus clustering enhances the robustness and stability of spatial domain identification.

To evaluate the effectiveness of STMVGAE, we tested it using five real ST datasets from three different platforms. Experimental results demonstrate that STMVGAE performs well across these datasets, exhibiting good performance in various downstream analyses including spatial domain identification, UMAP visualization, PAGA trajectory analysis, denoising, and batch integration.

We attribute the advantages of STMVGAE to several factors. Firstly, it leverages gene expression data along with histological images, effectively improving model performance. Secondly, STMVGAE learns different neighbor relationships between spots by training on various adjacency matrices constructed using spatial coordinate information. Thirdly, the utilization of consensus clustering enhances the robustness and stability of the results.

As spatial transcriptomics (ST) technology continues to evolve, producing larger and higher resolution ST data, STMVGAE's limitation may lie in clustering prediction labels being utilized for spatial domain identification, while other downstream analyses require low-dimensional embedding representations. In future work, we aim to explore methods for integrating low-dimensional embeddings from multiple views.

## Code availability

The STMVGAE package is implemented in Python and is available at https://github.com/wenwenmin/STMVGAE.

## Funding

The work was supported in part by the 10.13039/501100001809National Natural Science Foundation of China (62262069), in part by the Yunnan Fundamental Research Project (202301AT070230), in part by the Program of Yunnan Key Laboratory of Intelligent Systems and Computing (202405AV340009) and Young Talent Program of Yunnan Province (C619300A067).

## CRediT authorship contribution statement

**Jinyun Niu:** Writing – original draft, Visualization, Validation, Methodology, Investigation, Formal analysis, Data curation. **Fangfang Zhu:** Writing – original draft, Visualization, Validation, Supervision, Resources, Methodology, Data curation. **Taosheng Xu:** Validation, Supervision, Project administration, Investigation. **Shunfang Wang:** Project administration, Investigation, Formal analysis, Conceptualization. **Wenwen Min:** Writing – review & editing, Supervision, Project administration, Investigation, Funding acquisition, Conceptualization.

## Declaration of Competing Interest

The authors declare that they have no known competing financial interests or personal relationships that could have appeared to influence the work reported in this paper.

## Data Availability

All datasets in this article are accessible from here: (1) 10x Visium human dorsolateral prefrontal cortex dataset: http://spatial.libd.org/spatialLIBD/; (2) 10x Visium human breast cancer dataset: https://www.10xgenomics.com/datasets/human-breast-cancer-block-a-section-1-1-standard-1-1-0; (3) 10x Visium Human breast cancer: ductal carcinoma in situ dataset: https://www.10xgenomics.com/datasets/human-breast-cancer-ductal-carcinoma-in-situ-invasive-carcinoma-ffpe-1-standard-1-3-0; (4) sptial-research melanoma cancer dataset: https://github.com/1alnoman/ScribbleDom; (5) Stereo-seq mouse olfactory bulb dataset: https://github.com/JinmiaoChenLab/SEDR_analyses. (6) The ISH images of the adult human brain in gene denosing task are available at the Allen Human Brain Atlas: https://human.brain-map.org/.

## Appendix A Supplementary material

MMC The supplementary file provides a detailed description including comparison methods, performance metrics, the experimental setup used in this study, and other experimental results. MMC
