# Supplementary material for: Deep clustering representation of spatially resolved transcriptomics data using multi-view variational graph auto-encoders with consensus clustering
Source: Comput Struct Biotechnol J. 2024 Dec 2;23:4369–83. doi: 10.1016/j.csbj.2024.11.041 (PMC11664090; doi:10.1016/j.csbj.2024.11.041)
Supplement: MMC — The supplementary file provides a detailed description including comparison methods, performance metrics, the experimental setup used in this study, and other experimental results. [file mmc1.pdf]

Supplementary Materials

1. Supplementary Note

1.1. *Comparison with other spatial domain identification methods parameter settings*

We quantitatively compared STMVGAE with other methods on different datasets, including the non-spatial method SCANPY [1], and the spatial methods stLearn [2], SEDR [3], SpaGCN [4], DeepST [5], STAGATE [6] and STAMaker [7].

The selection of these comparison methods was based on the following considerations:
1.Relevance of methods. The chosen methods are advanced approaches specifically designed for spatial domain identification tasks in spatial transcriptomics. They address key research objectives, including spatial domain recognition, gene expression pattern analysis, and spatial data feature extraction, making them highly relevant to our study.
2. Influence in the Field. These methods have gained widespread recognition and citations in spatial transcriptomics research and have become benchmark models in the field.
3. Method Diversity. The selected methods encompass a variety of technical paradigms, enabling us to compare the performance of different types of approaches in spatial transcriptomics data analysis.

The parameter settings of these methods are as follows:

- SCANPY: First, we used the same data preprocessing method as STMVGAE to preprocess gene expression (log-transformed, normalized and selecting the top 3,000 HVGs). PCA dimensionality reduction was then used to reduce the gene expression data to 30 PCs. Finally, we used the *scanpy.pp.neighbor()* function default parameters provided by the SCNAPY package [1] to calculate neighbors, and the *scanpy.tl.louvain()* function is used to allocate spots. Additionally, the resolution parameter was tuned manually to ensure the number of clustering is equal to the ground truth.
- stLearn: We chose default parameters for stLearn on the DLPFC dataset. Specifically, the *stLearn.SME.SME_normalized()* function was performed on the raw gene expression of all genes with the parameter use_data=*"raw"*

and weights=*"physical_distance"*. Then the first 30 PCs of the SME normalized matrix were used for clustering. We did not use stLearn for training on the melanoma dataset because it does not support training without histology images.

- SEDR: SEDR can be trained on all datasets, and we retain all its default parameters except for empirically selecting the number of neighbors on different datasets to ensure reasonable results. We perform the same strategy on each dataset, looking for the number of neighbors that gives the best results between 6 and 12 neighbors. We set n in the *SEDR.graph_construction()* function to 10 on the DLPFC dataset and to 12 on all other datasets.
- SpaGCN: We use its recommended parameters for SpaGCN in all datasets.
- DeepST: We retain all the default parameters of DeepST and set k in the *deepen._get_graph()* function to 12. Additional, We tested the results on the melanoma dataset with DeepST set up without using histological images.
- STAGATE: STAGATE builds the graph by looking for neighbors within a radius, so the parameter r in the *STAGATE.Cal_Spatial_Net()* function changes in each dataset. We used the same rules as SEDR to select r. In DLPFC, we used the recommended parameter r set to 150, r in the BCDC data set to 350, r in the melanoma data set to 2, and r in the BRCA data set to 300.
- STAMaker: Recommended parameters are used in STAMaker, and neighbor selection is consistent with STAGATE. We set n to randomly initialize the model in STAMaker to 5.

1.2. ***Evaluation metrics of clustering***

ARI. The adjusted Rand index (ARI) [8] is a measure of the similarity between two clusterings, and it is an external evaluation index. We introduce ARI to calculate the similarity between the results obtained by STMVGAE spot assignment and manual annotation. The calculation of ARI must first calculate the values of the contingency table. The contingency table contains the following four parts: TF is the count of spot pairs classified into the same cluster in both the true and predicted clustering. TN is the count of spot pairs classified into different clusters in both the true and predicted clustering. FN is the count of spot pairs classified into the same cluster

in the true clustering but into different clusters in the predicted clustering. FP is the count of spot pairs classified into different clusters in the true clustering but into the same cluster in the predicted clustering. The value range of ARI is between [-1,1]. Generally, the closer the ARI value is to 1, the better the result. The closer the ARI value is to 0, the clustering result is the same as the random clustering result. The calculation method of ARI is based on paired samples. It considers the combination of samples of the same category in different clusters in two clustering results and compares it with random situations. ARI is computed as:

$$ARI = \frac{TP + TN - E}{TP + TN + FP + FN - E} \tag{1}$$

The expected value of the index, denoted as E, represents the value that would be obtained if the clustering were entirely random. It is calculated as follows:

$$E = \frac{(TP + FP) \times (TP + FN) + (FN + TN) \times (FP + TN)}{TP + TN + FP + FN} \tag{2}$$

NMI. Normalized Mutual Information (NMI) is an indicator used to evaluate the performance of clustering algorithms. It measures the similarity between two clustering results. The NMI value ranges between [0,1]. The closer the value is to 1, the more similar the two clustering results are, while the closer the value is to 0, the less similar they are. P represents the spatial domain clustering result and T represents the ground truth clustering labels. Their entropies are denoted as $H(P)$ and $H(T)$ respectively. NMI has been widely used to evaluate the performance of spatial domain identification in spatial transcriptomic data analysis [9]. The calculation formula for NMI is as follows:

$$NMI = \frac{MI(P,T)}{\sqrt{H(P)H(T)}} \tag{3}$$

HS. In unsupervised clustering, Homogeneity Score (HS) is a metric used to evaluate clustering results, which measures whether the samples in each cluster belong to the same category [6]. The value of HS ranges from 0 to 1. The closer the value is to 1, the better the clustering result is, that is, each cluster contains samples of the same category. $H(C)$ is the entropy of the true class, which represents the uncertainty of the class distribution of

the samples in the data set; $H(C|K)$ is the conditional category entropy of a given clustering result, which represents the uncertainty of the category distribution of the sample when the clustering result is known. The calculation formula for HS is as follows:

$$HS = 1 - \frac{H(C|K)}{H(C)} \tag{4}$$

Purity. In unsupervised clustering, Purity is a metric used to evaluate clustering results, which measures whether the samples contained in each cluster belong to the same category. The value range of Purity is between 0 and 1. The closer the value is to 1, the better the clustering result is, that is, each cluster contains samples of the same category. N is the total number of samples in the dataset, k represents the index of the cluster, j represents the index of the real category, c_k represents the sample set in cluster k, and t_j represents the sample set in real category j. The $|c_k \cap t_j|$ in the formula represents the size of the intersection of samples in cluster k and samples in true category j. The calculation formula for Purity is as follows:

$$Purity = \frac{1}{N} \sum_k max_j |c_k \cap t_j| \tag{5}$$

1.3. ***Implementation Details***

Our experiments were performed on a single NVIDIA RTX 4090Ti GPU using PyTorch (version 1.13.1) and Python 3.11. In the "ST data augmentation" section, we selected Resnet50 as the default convolutional neural network, and then introduced two parameters α_1 and α_2 to balance the image feature matrix and the gene expression matrix. We fixed α_2 to 1.0, and set α_1 to 0.2 through experiments. In the "Spatial graph construction" section, we selected neighbors with a strategy that the number of neighbors will not be zero and will not generate too many neighbors (more than 12), and the best performance is obtained within this range. The baseline method also used the same strategy. On different datasets, the construction parameters of the adjacency matrix are set as follows:

- DLPFC dataset: adjacency matrix parameters based on Radius $r = 250$, adjacency matrix parameters based on KNN $k = 12$.
- BCDC dataset: adjacency matrix parameters based on Radius $r = 300$, adjacency matrix parameters based on KNN $k = 5$.

- Melanoma dataset: adjacency matrix parameters based on Radius $r = 3$, adjacency matrix parameters based on KNN $k = 7$.
- BRCA dataset: adjacency matrix parameters based on Radius $r = 500$, adjacency matrix parameters based on KNN $k = 11$.

The linear encoder was set to [1000, 400, 30]. Then, the hidden representation is learned through a two-layer GCN encoder. The GCN waw set to [64, 8] and the linear decoder is set to [400, 1000]. The training strategy was established for 1000 epochs, the learning rate set at 0.001 and the weight decay set at 0.0001. For the loss function, we chose the hyperparameters empirically, we set λ_1 and λ_4 to 0.1, λ_2 and λ_3 to 1.0.

138 ## 2. Supplementary Figure

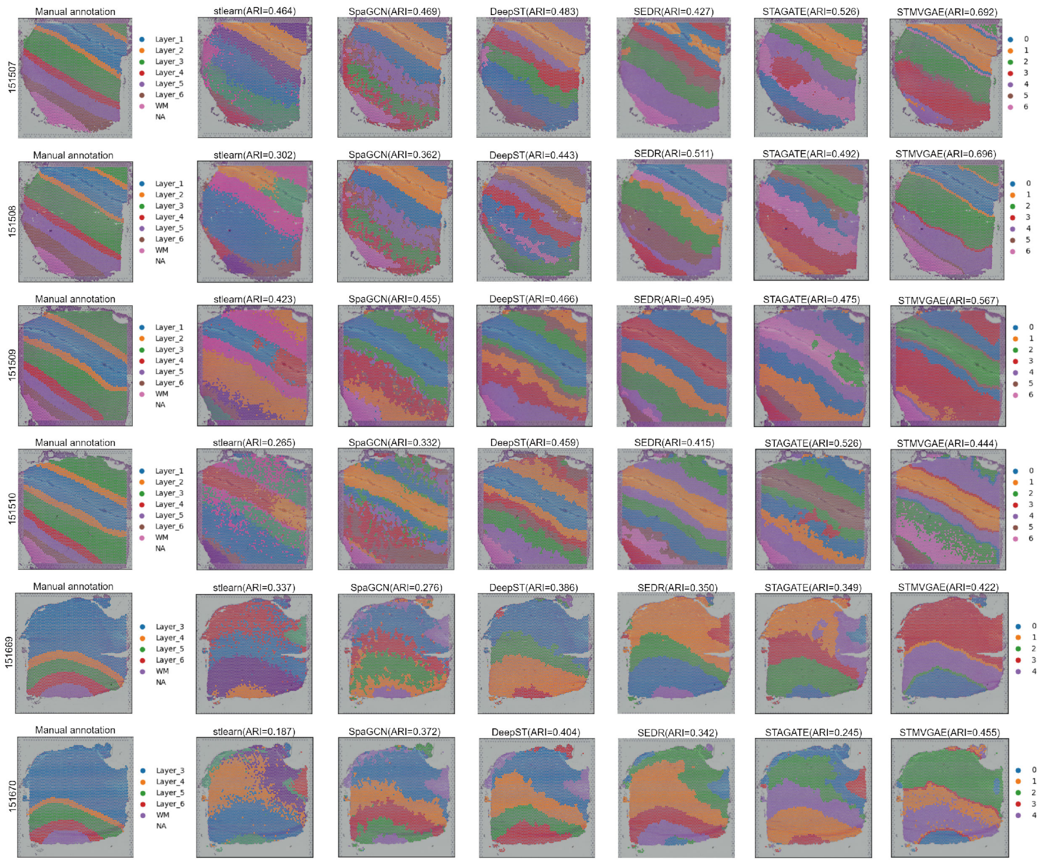

See next page

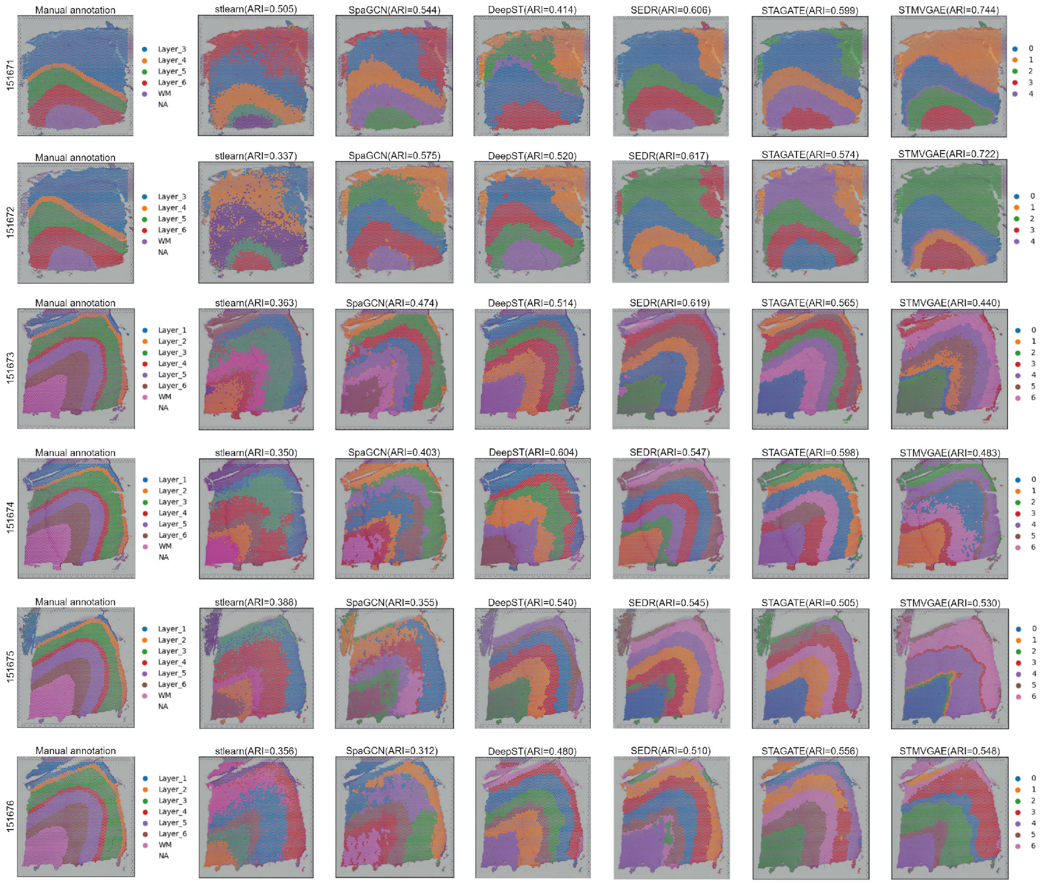

Figure S1. Comparison of spatial domains identification by clustering assignments via STMVGAE, STAGATE, SEDR, DeepST, stLearn, and manual annotation in all 12 slices of the DLPFC dataset.

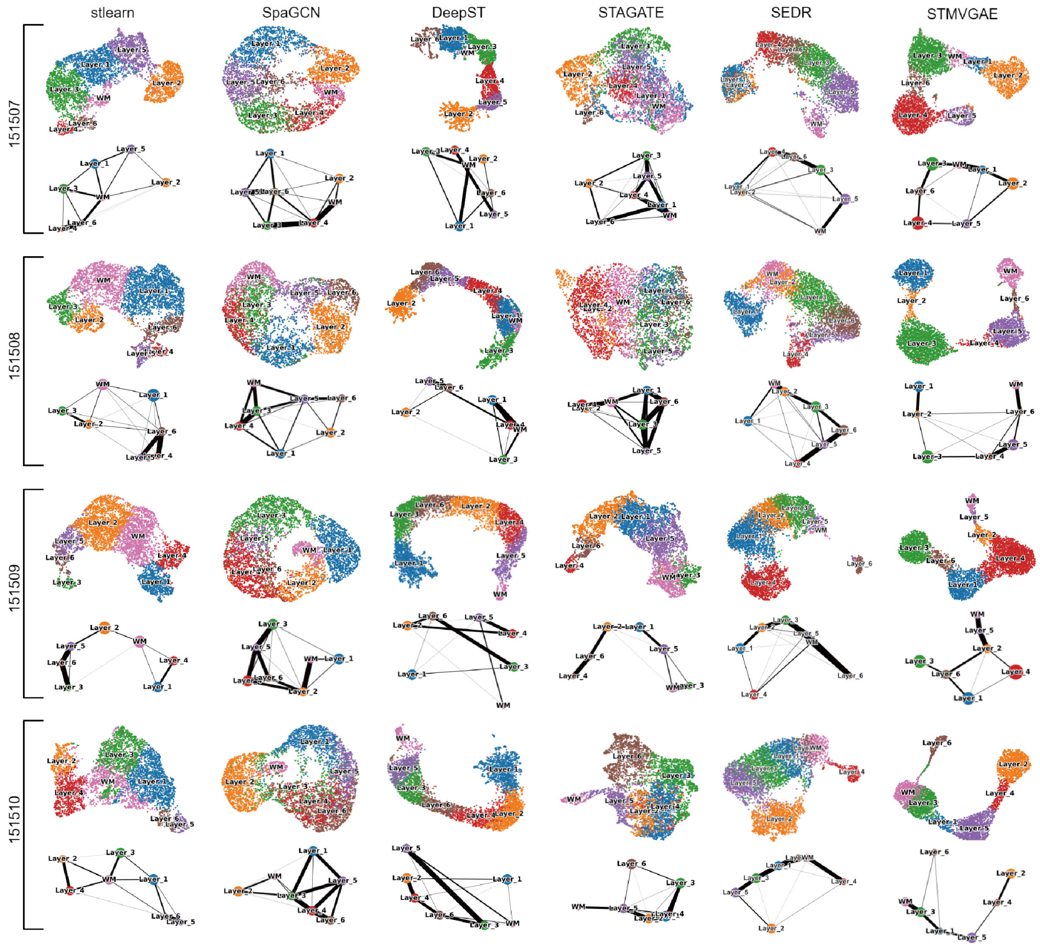

See next page

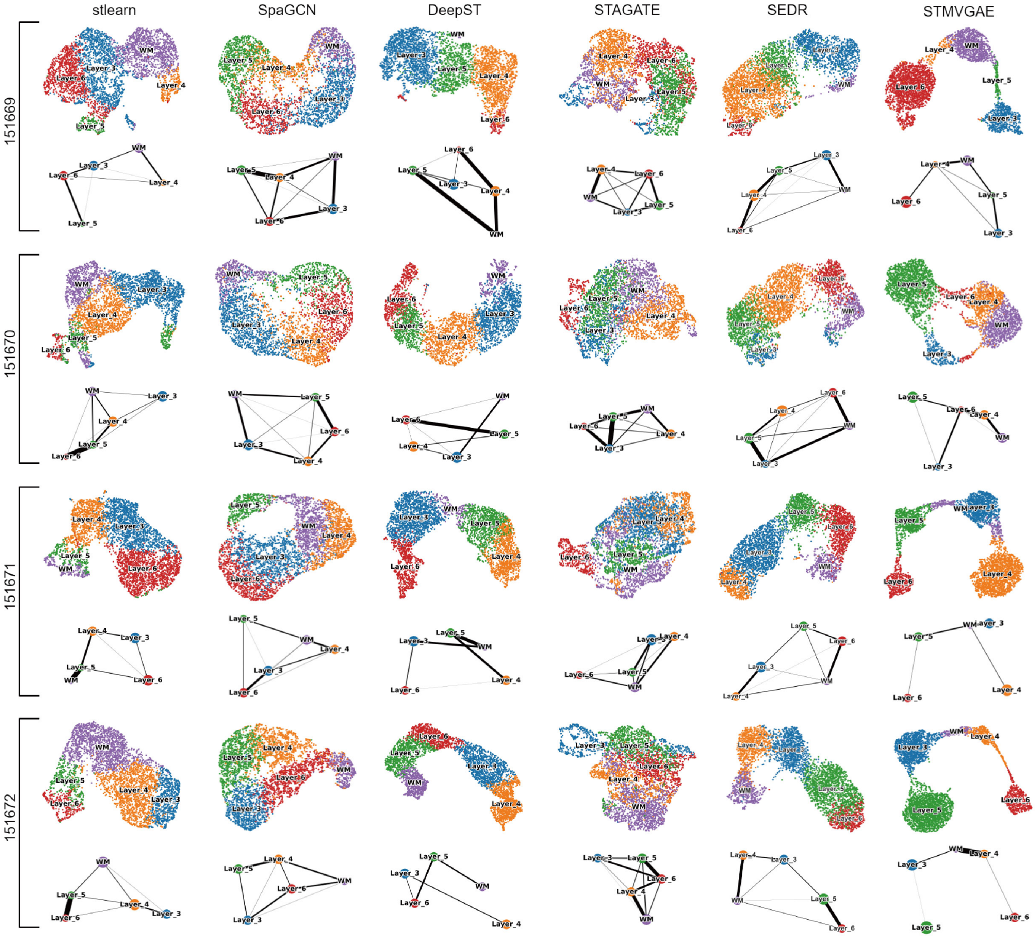

See next page

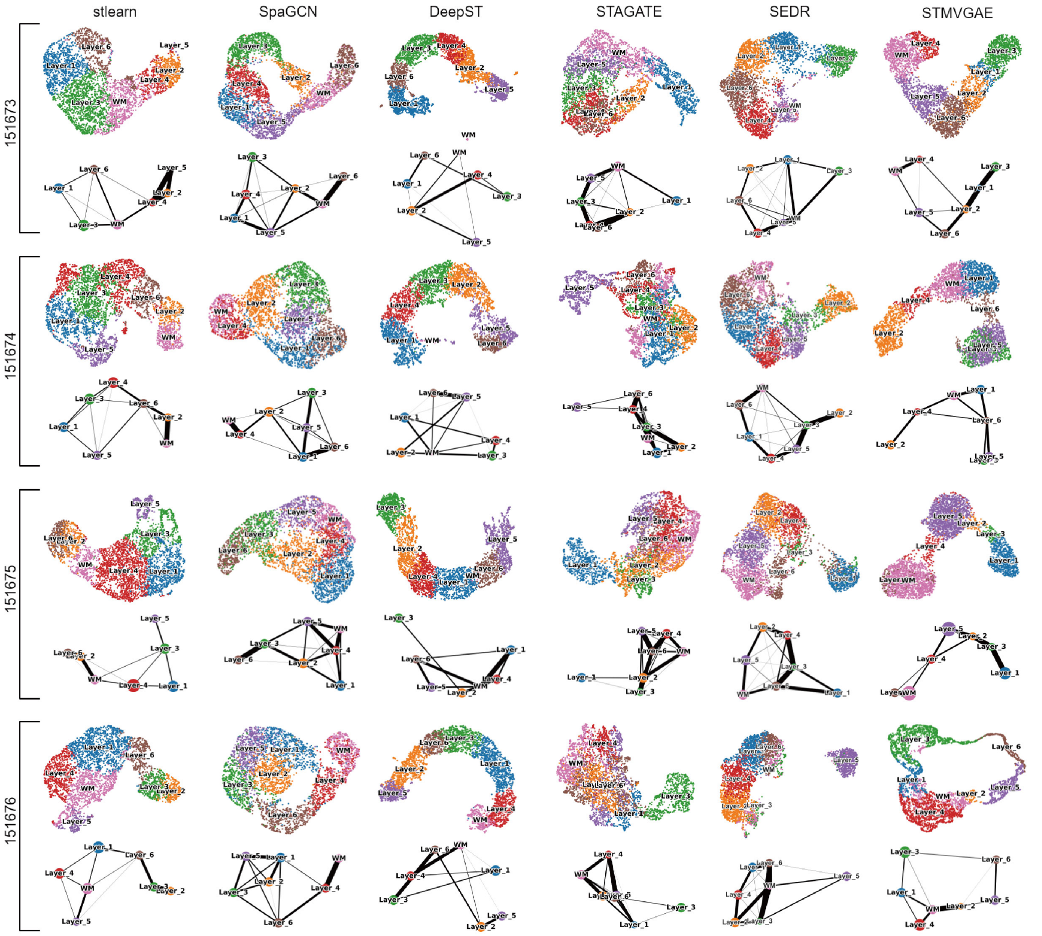

Figure S2. UMAP visualization and PAGA trajectory inference by STMVGAE, SEDR, STAGATE, DeepST, SpaGCN, and stLearn embeddings respectively.

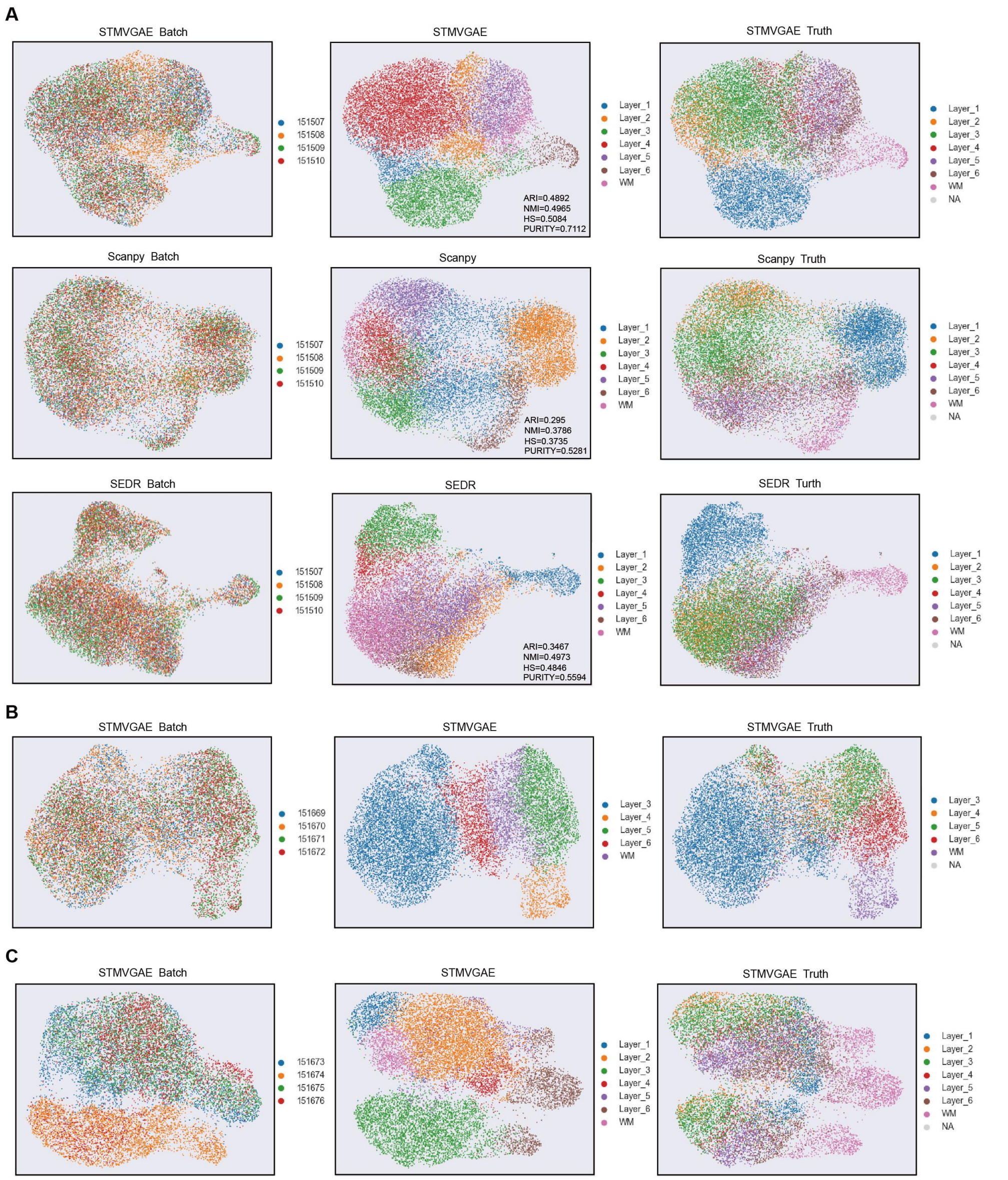

Figure S3. (**A**) UMAP visiulization of multi-slice joint analysis on 151507-151510 slices in DLPFC datasets. Each row represents the use of STMVGAE, SCANPY, and SEDR methods with Harmony for batch integration, and each column represents batches, identification spatial domains, and ground truth labels, respectively. (**B**) STMVGAE performs multi-slice joint analysis on 151669-151672 slices in the DLPFC dataset. (**C**) STMVGAE performs multi-slice joint analysis on 151673-151676 slices in the DLPFC dataset.

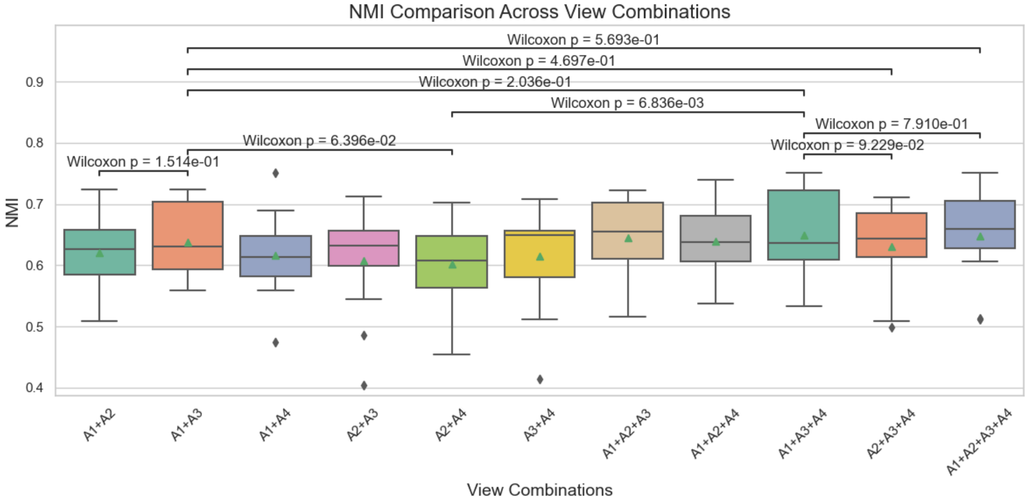

Figure S4. Box plot and significance markers of the NMI values of the STMVGAE method under multiple view combinations. The significance markers are calculated by the Wilcoxon rank sum test.

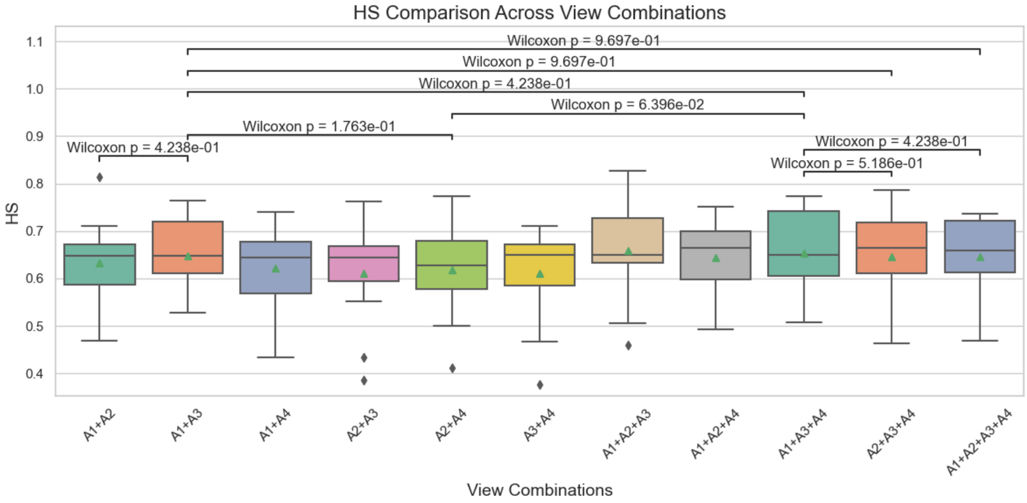

Figure S5. Box plot and significance markers of the HS values of the STMVGAE method under multiple view combinations. The significance markers are calculated by the Wilcoxon rank sum test.

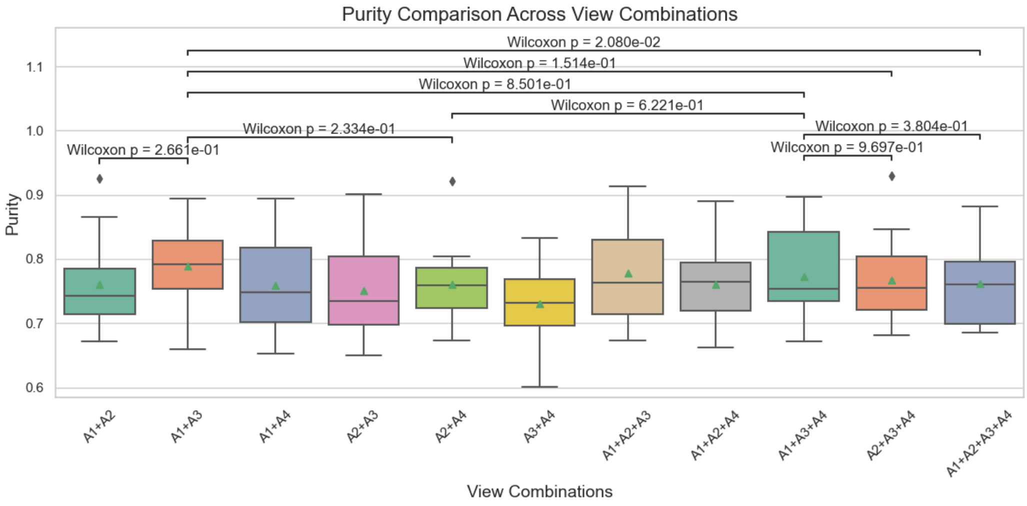

Figure S6. Box plot and significance markers of the Purity values of the STMVGAE method under multiple view combinations. The significance markers are calculated by the Wilcoxon rank sum test.

3. Supplementary Table

Table S1: Overview of comparative spatial domain identification methods.

Method	Methodology	Input Data	Downstream tasks	Link
SCANPY [1]	Non-spatial<br>method	Gene expression data	Spatial domain identification<br>Visualization<br>Trajectory inference	https://scanpy.<br>readthedocs.io/
stLearn [2]	Deep neural<br>network	Gene expression data<br>Histology information	Spatial domain identification<br>Visualization<br>Trajectory inference	https://github.com/<br>Biomedical<br>MachineLearning/<br>stLearn
SEDR [3]	Variational graph<br>autoencoders	Spatial location data<br>Gene expression data	Spatial domain identification<br>Visualization<br>Trajectory inference<br>Denosing<br>Batch integration	https://github.com/<br>HzFu/SEDR/
SpaGCN [4]	Graph convolutional<br>networks	Spatial location data<br>Gene expression data<br>Histology information	Spatial domain identification<br>Visualization<br>Trajectory inference<br>SVGs identification	https://github.com/<br>jianhuupenn/SpaGCN/
DeepST [5]	Variational graph<br>autoencoders	Spatial location data<br>Gene expression data<br>Histology information	Spatial domain identification<br>Visualization<br>Trajectory inference<br>Batch integration	https://github.com/<br>JiangBioLab/DeepST/
STAGATE [6]	Graph attention<br>autoencoders	Spatial location data<br>Gene expression data	Spatial domain identification<br>Visualization<br>Trajectory inference<br>Denosing	https://github.com/<br>zhanglabtools/STAGATE/

Table S2: Summary of the datasets in this study.

Platform	Tissue	Section	Number of domains	Spots	Genes
10X Visium	Human dorsolateral prefrontal cortex (DLPFC)[10]	151507	7	4226	33538
		151508	7	4384	
		151509	7	4789	
		151510	7	4634	
		151669	5	3661	
		151670	5	3498	
		151671	5	4110	
		151672	5	4015	
		151673	7	3639	
		151674	7	3673	
		151675	7	3592	
		151676	7	3460	
	Human breast cancer: ductal carcinoma in situ[11]	\	2	2518	17943
	Human breast[3] cancer	\	20	3798	36601
Spatialresearch	Melanoma cancer[12]	\	4	293	16148
Stereo-seq	Mouse olfactory bulb[13]	\	\	19109	27106

Table S3: Experiments on selection of hyperparameters α_1 and α_2. Because gene expression is important for spatial transcriptomics analysis, we want to preserve its raw gene expression matrix, so we fixed α_2 to 1.0. We explored the impact of the "ST data augmentation" module on performance by changing α_1.

α_1	DLPFC				BCDC				BRCA			
	ARI	NMI	HS	Purity	ARI	NMI	HS	Purity	ARI	NMI	HS	Purity
1.0	0.261	0.384	0.377	0.540	0.523	0.415	0.393	0.865	0.535	0.635	0.626	0.583
0.5	0.437	0.592	0.614	0.752	0.677	0.572	0.547	0.914	0.569	0.682	0.671	0.634
0.2	**0.562**	**0.638**	**0.648**	**0.789**	**0.730**	**0.584**	**0.583**	**0.931**	**0.660**	**0.699**	**0.689**	**0.678**

Table S4: STMVGAE performs graph combination test results on 12 slices of the DLPFC dataset. STMVGAE integrates the results of four different graphs in a free combination manner to calculate ARI, NMI, HS, and Pur (Purity) respectively. $A^{(1)}$, $A^{(2)}$, $A^{(3)}$, and $A^{(4)}$ represent Radius_balltree, Radius_kdtree, KNN_balltree, and KNN_kdtree respectively. The best result is underlined.

Slice	$A^{(1)} + A^{(2)}$				$A^{(1)} + A^{(3)}$				$A^{(1)} + A^{(4)}$				$A^{(2)} + A^{(3)}$				$A^{(2)} + A^{(4)}$				$A^{(3)} + A^{(4)}$			
	ARI	NMI	HS	Pur	ARI	NMI	HS	Pur	ARI	NMI	HS	Pur	ARI	NMI	HS	Pur	ARI	NMI	HS	Pur	ARI	NMI	HS	Pur
151507	0.549	0.662	0.664	0.685	0.692	0.712	0.763	0.860	0.548	0.644	0.658	0.737	0.561	0.677	0.675	0.698	0.501	0.648	0.673	0.754	0.567	0.698	0.710	0.750
151508	0.594	0.657	0.681	0.813	0.696	0.703	0.724	0.821	0.666	0.689	0.739	0.841	0.582	0.620	0.640	0.801	0.503	0.604	0.606	0.691	0.573	0.664	0.654	0.686
151509	0.421	0.585	0.573	0.672	0.567	0.644	0.636	0.783	0.421	0.588	0.581	0.704	0.504	0.637	0.609	0.699	0.411	0.567	0.560	0.673	0.604	0.653	0.643	0.773
151510	0.557	0.651	0.610	0.737	0.444	0.562	0.532	0.660	0.403	0.559	0.530	0.653	0.548	0.651	0.607	0.719	0.496	0.648	0.613	0.734	0.410	0.560	0.544	0.648
151669	0.400	0.523	0.513	0.775	0.422	0.570	0.530	0.739	0.415	0.562	0.527	0.776	0.201	0.405	0.386	0.670	0.375	0.492	0.499	0.769	0.342	0.512	0.467	0.701
151670	0.386	0.509	0.468	0.722	0.455	0.559	0.527	0.758	0.337	0.475	0.433	0.693	0.324	0.486	0.433	0.650	0.314	0.455	0.412	0.688	0.246	0.414	0.376	0.601
151671	0.770	0.724	0.711	0.866	0.744	0.707	0.720	0.895	0.784	0.751	0.741	0.894	0.706	0.688	0.665	0.833	0.746	0.703	0.773	0.921	0.698	0.708	0.688	0.833
151672	0.670	0.697	0.814	0.925	0.722	0.724	0.740	0.851	0.640	0.658	0.690	0.811	0.700	0.713	0.762	0.902	0.686	0.701	0.710	0.805	0.617	0.654	0.692	0.831
151673	0.446	0.624	0.670	0.731	0.440	0.618	0.639	0.708	0.464	0.638	0.659	0.739	0.430	0.618	0.647	0.744	0.496	0.645	0.699	0.804	0.499	0.647	0.656	0.749
151674	0.454	0.584	0.591	0.689	0.483	0.608	0.655	0.801	0.427	0.607	0.610	0.667	0.458	0.544	0.552	0.725	0.420	0.554	0.584	0.743	0.466	0.588	0.599	0.711
151675	0.504	0.628	0.656	0.764	0.530	0.601	0.641	0.817	0.538	0.621	0.674	0.839	0.479	0.629	0.697	0.815	0.486	0.601	0.641	0.764	0.528	0.652	0.665	0.767
151676	0.444	0.601	0.641	0.750	0.548	0.643	0.667	0.773	0.513	0.602	0.632	0.758	0.469	0.634	0.664	0.762	0.488	0.611	0.652	0.780	0.477	0.632	0.642	0.715
Average	0.516	0.620	0.633	0.761	0.562	0.638	0.648	0.789	0.513	0.616	0.623	0.759	0.497	0.608	0.611	0.751	0.494	0.602	0.618	0.761	0.502	0.615	0.611	0.730

Table S5: STMVGAE performs graph combination test results on 12 slices of the DLPFC dataset. STMVGAE integrates the results of four different graphs in a free combination manner to calculate ARI, NMI, HS, and Pur (Purity) respectively. $A^{(1)}$, $A^{(2)}$, $A^{(3)}$, and $A^{(4)}$ represent Radius_balltree, Radius_kdtree, KNN_balltree, and KNN_kdtree respectively. The best result is underlined.

Slice	$A^{(1)} + A^{(2)} + A^{(3)}$				$A^{(1)} + A^{(2)} + A^{(4)}$				$A^{(1)} + A^{(3)} + A^{(4)}$				$A^{(2)} + A^{(3)} + A^{(4)}$				$A^{(1)} + A^{(2)} + A^{(3)} + A^{(4)}$			
	ARI	NMI	HS	Pur	ARI	NMI	HS	Pur	ARI	NMI	HS	Pur	ARI	NMI	HS	Pur	ARI	NMI	HS	Pur
151507	0.618	0.708	0.827	0.913	0.581	0.699	0.710	0.761	0.688	0.729	0.773	0.866	0.569	0.700	0.715	0.771	0.583	0.704	0.719	0.776
151508	0.660	0.700	0.721	0.824	0.625	0.674	0.696	0.814	0.705	0.723	0.753	0.839	0.597	0.681	0.724	0.847	0.676	0.708	0.730	0.821
151509	0.570	0.657	0.641	0.776	0.428	0.612	0.601	0.693	0.570	0.646	0.630	0.747	0.496	0.643	0.618	0.696	0.574	0.656	0.644	0.777
151510	0.501	0.652	0.635	0.717	0.438	0.634	0.603	0.680	0.411	0.588	0.563	0.678	0.421	0.622	0.593	0.681	0.433	0.635	0.614	0.686
151669	0.353	0.543	0.506	0.746	0.417	0.551	0.515	0.773	0.374	0.558	0.534	0.739	0.384	0.498	0.495	0.767	0.298	0.513	0.481	0.727
151670	0.335	0.516	0.459	0.673	0.423	0.537	0.493	0.741	0.481	0.534	0.507	0.764	0.379	0.509	0.462	0.685	0.443	0.512	0.470	0.743
151671	0.745	0.723	0.748	0.910	0.826	0.740	0.751	0.890	0.790	0.751	0.742	0.896	0.729	0.701	0.785	0.929	0.798	0.751	0.735	0.882
151672	0.726	0.718	0.755	0.848	0.704	0.705	0.748	0.827	0.725	0.723	0.742	0.855	0.709	0.712	0.728	0.815	0.718	0.717	0.736	0.850
151673	0.500	0.654	0.659	0.694	0.543	0.675	0.694	0.788	0.532	0.672	0.687	0.741	0.498	0.645	0.648	0.738	0.512	0.665	0.665	0.702
151676	0.490	0.592	0.625	0.749	0.422	0.587	0.590	0.662	0.477	0.628	0.620	0.672	0.460	0.592	0.625	0.742	0.463	0.607	0.613	0.693
151675	0.528	0.662	0.691	0.779	0.507	0.624	0.652	0.768	0.462	0.616	0.655	0.760	0.473	0.621	0.680	0.801	0.531	0.661	0.693	0.788
151676	0.442	0.616	0.635	0.708	0.471	0.643	0.679	0.728	0.445	0.616	0.644	0.721	0.479	0.647	0.687	0.730	0.476	0.642	0.654	0.691
Average	0.539	0.645	0.658	0.778	0.532	0.640	0.644	0.760	0.555	0.649	0.654	0.773	0.516	0.631	0.647	0.767	0.542	0.648	0.646	0.761

References

[1] F Alexander Wolf, Philipp Angerer, et al. SCANPY: large-scale single-cell gene expression data analysis. *Genome Biology*, 19:1–5, 2018.

[2] Duy Pham, Xiao Tan, et al. stlearn: Robust mapping of spatiotemporal trajectories and cell–cell interactions in healthy and diseased tissues. *Nature Communications*, 14(1):7739, 2023.

[3] Hang Xu, Huazhu Fu, et al. SEDR: Unsupervised spatially embedded deep representation of spatial transcriptomics. *Genome Medicine*, 16(1):1–15, 2024.

[4] Jian Hu, Xiangjie Li, et al. SpaGCN: Integrating gene expression, spatial location and histology to identify spatial domains and spatially variable genes by graph convolutional network. *Nature Methods*, 18(11):1342–1351, 2021.

[5] Chang Xu, Xiyun Jin, et al. DeepST: identifying spatial domains in spatial transcriptomics by deep learning. *Nucleic Acids Research*, 50(22):e131–e131, 2022.

[6] Kangning Dong and Shihua Zhang. STAGATE: Deciphering spatial domains from spatially resolved transcriptomics with an adaptive graph attention auto-encoder. *Nature Communications*, 13(1):1739, 2022.

[7] Chihao Zhang, Kangning Dong, et al. STAMarker: determining spatial domain-specific variable genes with saliency maps in deep learning. *Nucleic Acids Research*, 51(20):e103–e103, 2023.

[8] Lawrence Hubert and Phipps Arabie. Comparing partitions. *Journal of Classification*, 2:193–218, 1985.

[9] Zhiyuan Yuan, Fangyuan Zhao, Senlin Lin, Yu Zhao, Jianhua Yao, Yan Cui, Xiao-Yong Zhang, and Yi Zhao. Benchmarking spatial clustering methods with spatially resolved transcriptomics data. *Nature methods*, 21(4):712–722, 2024.

[10] Kristen R Maynard, Leonardo Collado-Torres, et al. Transcriptome-scale spatial gene expression in the human dorsolateral prefrontal cortex. *Nature Neuroscience*, 24(3):425–436, 2021.

[11] Mohammad Nuwaisir Rahman, Abdullah Al Noman, et al. ScribbleDom: using scribble-annotated histology images to identify domains in spatial transcriptomics data. *Bioinformatics*, 39(10):btad594, 2023.

[12] Kim Thrane, Hanna Eriksson, et al. Spatially resolved transcriptomics enables dissection of genetic heterogeneity in stage iii cutaneous malignant melanoma. *Cancer Research*, 78(20):5970–5979, 2018.

[13] Ao Chen, Sha Liao, et al. Spatiotemporal transcriptomic atlas of mouse organogenesis using dna nanoball-patterned arrays. *Cell*, 185(10):1777–1792, 2022.
